# Aerosol Uptake Coefficients of Isoprene Epoxides: Determination and Parameter Estimation from Online Field Measurements of Organic Molecular Markers

**DOI:** 10.1021/acs.est.5c09046

**Published:** 2025-08-22

**Authors:** Shuhui Zhu, Jie Zhang, Li Li, Min Zhou, Liping Qiao, Hongli Wang, Dan Dan Huang, Qiongqiong Wang, Shengao Jing, Yuhang Wu, Shan Wang, Changhong Chen, Qi Ying, Jian Zhen Yu

**Affiliations:** † State Environmental Protection Key Laboratory of Formation and Prevention of Urban Air Pollution Complex, 177494 Shanghai Academy of Environmental Sciences, Shanghai 200233, China; ‡ Division of Environment and Sustainability, 58207 Hong Kong University of Science and Technology, Kowloon 999077, China; § Zachary Department of Civil and Environmental Engineering, Texas A&M University, College Station, Texas 77843-3136, United States; ∥ Rural Environment Protection Engineering & Technology Center of Sichuan Province, College of Environmental Sciences, 506176 Sichuan Agricultural University, Chengdu 611830, China; ⊥ Department of Atmospheric Science, School of Environmental Studies, China University of Geosciences, Wuhan 430074, China; # Department of Chemistry, 58207 Hong Kong University of Science and Technology, Kowloon 999077, China

**Keywords:** isoprene SOA, isoprene epoxides, heterogeneous reaction rates, uptake coefficients, aqueous-phase rate constants

## Abstract

This study introduces a new approach that combines online field measurements and photochemical box modeling to estimate summer daytime heterogeneous reaction rates (*k*_het_) and uptake coefficients (γ) for isoprene epoxydiols (IEPOX) and hydroxymethyl-methyl-α-lactone (HMML)/methacrylic acid epoxide (MAE) under ambient conditions. The *k*_het_ and γ values are highly acidity-dependent. For IEPOX, γ increases from (2.1 ± 1.5) × 10^–3^ under pH > 4 to (6.7 ± 6.0) × 10^–3^ under pH < 2, and *k*_het_ increases from (2.2 ± 1.3) × 10^–5^ under pH > 4 to (7.3 ± 6.7) × 10^–5^ under pH < 2. For HMML/MAE, γ increases from (2.2 ± 1.9) × 10^–5^ under pH > 4 to (1.3 ± 1.1) × 10^–4^ under pH < 2, and *k*_het_ increases from (2.3 ± 1.9) × 10^–7^ under pH > 4 to (1.4 ± 1.3) × 10^–6^ under pH < 2. These estimates are comparable to those derived from chamber experiments under similar conditions but higher than those from field studies estimated by reactive uptake parametrization. Furthermore, multilinear regression is employed to determine three aqueous phase reaction parameters ( $H_{aq}k_{{H_{2}O,H}^{+}}$ , $H_{aq}k_{H_{2}O,{HSO}_{4}^{-}}$ and $H_{aq}k_{{{\text{SO}}_{4}^{2-},H}^{+}}$ ). These parameters can be used to estimate γ in chemical transport models, and their values approach the upper limit of previously reported data. The study demonstrates the feasibility of deriving kinetic parameters for isoprene SOA intermediates under ambient conditions using bihourly organic molecular tracer data.

## Graphical abstract

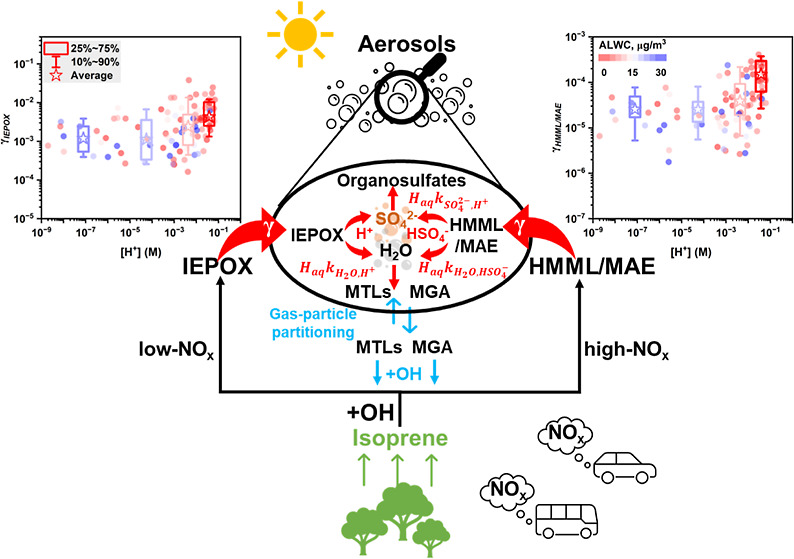

## 1 Introduction

Isoprene (C_5_H_8_), predominantly emitted by plants, contributes to more than 50% of global nonmethane hydrocarbon emissions.^1^ Early research^2^ suggested that the formation of secondary organic aerosol (SOA) from isoprene was insignificant under ambient conditions because its first-generation products, such as methacrolein (MACR) and methyl vinyl ketone (MVK), are highly volatile. Later, smog chamber experiments and field research have identified that isoprene epoxydiol (IEPOX) is a key intermediate of isoprene photooxidation under low-NO_*x*_ conditions. Meanwhile, methacrylic acid epoxide (MAE) and hydroxymethyl-methyl-α-lactone (HMML) are two major intermediates formed under the high NO_*x*_ conditions.^3–5^ These epoxides can enter aerosol aqueous phase via reactive surface uptake and subsequently form substantial amounts of SOA via the acid-driven nucleophilic reactions.^5^ Among the resulting SOA products are organosulfate compounds, which are formed when sulfate (SO_4_^2–^) acts as the nucleophile, and two specific marker compounds, 2-methyltetrols (MTLs) derived from IEPOX, and 2-methylglyceric acid (MGA), derived from HMML/MAE, when the nucleophile is the H_2_O molecule.

The pseudo-first order heterogeneous reaction rate coefficient (*k*_het_), which is closely related to the reactive uptake coefficients (γ), is used to quantify the rate of reactive surface uptake due to the combined influences of gas-to-particle diffusion, mass accommodation, dissolution, and aqueous diffusion and reactions. Although laboratory-derived *k*_het_ and γ values for IEPOX and HMML/MAE have been reported in several publications using entrained gas-aerosol flow reactor techniques,^6,7^ no attempt has been made to determine *k*_het_ and γ values based on the field measurements. Resistors-in-series parametrizations are commonly used to predict *k*_het_ and γ values for the formation of MTLs and MGA in ambient air. These parametrizations are derived from theoretical analyses of reactive mass transfer to submicron spherical particles.^6,7^ In such models, several key parameters, including aqueous phase reaction rate coefficients and Henry’s Law constants are determined using bulk solutions or empirically estimated. The reported values of these parameters from various experiments exhibit wide ranges of 1–3 orders of magnitude,^8–12^ resulting in significant uncertainties in the calculated *k*_het_ and γ values.

In this study, a new approach that integrates field measurements of particle phase MGA and MTLs (2-methylthreitol and 2-methylerythritol) and photochemical box modeling is applied to study the reactive uptake processes of IEPOX and HMML/MAE onto aerosols at an urban site in Shanghai. Using gas and aerosol data collected in the field and from photochemical modeling, we estimated the γ and *k*_het_ of IEPOX and HMML/MAE and derived a set of partitioning and reaction parameters that can be used in chemical transport models to estimate γ. These field-based parameters could potentially reduce uncertainties and improve accuracy in assessments of isoprene SOA impacts in regional and global chemical transport models.

## 2 Methods

### 2.1 Online Measurements

Bihourly measurements of MGA, 2-methylthreitol, and 2-methylerythritol were conducted at the Shanghai Academy of Environmental Sciences (SAES) in urban Shanghai (31°10′N,121°25′E) from March to December 2020 using a thermal desorption aerosol gas chromatography–mass spectrometry system (TAG, Aerodyne Research Inc.). Among these isoprene SOA tracers, 2-methylthreitol and 2-methylerythritol (MTLs) were quantified with authentic standard synthesized in the laboratory,^13^ and MGA was quantified by a surrogate approach using the calibration curve built for MTLs. More detailed information related to data quantification, uncertainty analysis, and quality control is provided in Text S1, while Table S1 summarizes the uncertainties associated with TAG measurements. A detailed description of the monitoring site and the TAG system can be found in previous publications.^14–16^

In addition to the aerosol-phase organic tracer measurements by the TAG system, a suite of other pollutants was concurrently monitored at the same site, which is briefly described below. A total of 139 VOCs, including isoprene, MACR, and MVK, were measured with a 30 min resolution using two online gas chromatography systems equipped with a flame ionization detector (GC-FID) (Chromato-sud airmoVOC C_2_–C_6_ and airmoVOC C_6_–C_12_, Chromatotec).^17,18^ Criteria pollutants, including PM_2.5_, NO_*x*_, and O_3_, were measured by an online beta attenuation particulate monitor (FH 62 C14 series, Thermo Fisher Scientific Inc.), a NO_*x*_ monitor (model 42i, Thermo Fisher Scientific Inc.), and an O_3_ monitor (model EC9811, Ecotech Inc.), respectively. A semicontinuous OC/EC analyzer (model RT-4, Sunset Laboratory) and a Monitor for AeRosols and Gases (MARGA, Applikon B.V.) were also deployed to provide hourly concentrations of carbonaceous species (OC and EC) and water-soluble inorganic ions (sulfate, nitrate, chloride, ammonium, sodium, potassium, calcium, and magnesium) in PM_2.5_, respectively.^19,20^ In addition, particle number size distributions ranging from 13.6 to 6335 nm were measured using a scanning mobility particle sizer (SMPS, TSI model 3772) and an aerodynamic particle sizer (APS, TSI model 3321), which were described in detail by Ling et al. (2019).^21^ For simulating concentrations of isoprene epoxides with photochemical box model, NO_2_ photolysis rates and planetary boundary layer (PBL) heights are also needed as input information. The photolysis frequencies of NO_2_ were measured by a high-resolution photolysis spectrometer (2pi-sr-CCD, Metcon) with 1 min time resolution.^22^ The hourly heights of PBL at the observation site were obtained from ERA5 reanalysis hourly data provided by European Centre for Medium-Range Weather Forecasts (ECMWF, https://cds.climate.copernicus.eu/cdsapp#!/dataset/reanalysis-era5-single-levels?tab=overview).

### 2.2 Observation-Based Modeling of Isoprene Epoxides

Gas-phase concentrations of the isoprene epoxides (IEPOX, HMML, and MAE) were simulated using an observation-based box model (OBM)^23^ developed based on the framework of the community multiscale air quality (CMAQ) model v5.0.1.^24–26^ The gas-phase chemical mechanism is based on SAPRC-11 (S11),^27^ and the original isoprene oxidation chemistry in the S11 mechanism was replaced by the expanded reactions described by Xie et al. (2014)^28^ and Lin et al. (2012).^5^ The modified mechanism has been applied in the previous modeling studies.^29–33^ In our OBM model, MAE is formed from the oxidation of MACR by OH radicals with a reduced yield of 0.02 based on the recent laboratory work of Schwantes et al. (2019),^34^ which differs from the original treatment where MAE is formed from the methacryloyl peroxy nitrate (MPAN) + OH reaction with a molar yield of 0.21. This update leads to reduced MAE formation from the high-NO_*x*_ pathway.

The hourly measured gas-phase species (i.e., O_3_, CO, SO_2_, NO, NO_2_, and VOCs) and meteorological parameters (i.e., temperature, relative humidity, wind speed, PBL height, and NO_2_ photolysis rate) were used as input data for the OBM. Epoxides were simulated based on all available input data and corrected for the contributions from regional transport (as shown in Figure S12). To be specific, the OBM adopted in this study focuses on local chemical processes only. We assumed that early generation oxidation products of isoprene (e.g., MACR and MVK) have less uncertainties in chemical reactions. Therefore, the amounts of epoxides from regional transport were estimated based on the relative differences of isoprene early generation oxidation products (i.e., MACR and MVK) between observations and model predictions that were simulated with the input data excluding MACR and MVK. The corrected hourly concentrations of IEPOX, HMML, and MAE are extracted to estimate *k*_het_ and γ. The performance of OBM was also evaluated by comparing modeled and observed concentrations of isoprene, MACR and MVK (Text S2). The modeled concentrations and temporal variations of MACR and MVK are generally in agreement with observations (Figure S8). In addition, the predicted particle phase IEPOX concentrations using the modeled gas-phase concentrations and parameters (e.g., *k*_het_, *k*_aq_, β_MTL‑OS_) derived from this study correlate well (*R* = 0.92) with the TAG-measured IEPOX concentrations (Figure S9). These results suggest that the chemical mechanisms included in the OBM model could well capture the major intermediate oxidation products of isoprene and the key precursor in the formation of isoprene SOA, providing confidence in the estimates of γ and *k*_het_. Furthermore, we also applied the bootstrap method to conduct sensitivity analysis of model results on the estimated coefficients. The bootstrap analysis results are given in Table S2. The mean values of the coefficients obtained from the bootstrap resampling are generally close to the original estimates reported in Table 2, suggesting that systematic errors introduced by OBM in *k*_het_ and γ estimations are likely limited.

### 2.3 Estimations of *K*_het_ and γ for Isoprene Epoxides

While nearly a year of online measurements were performed at this site, only summer (July–September) daytime data were selected for *k*_het_ and γ estimations. The contributions of regional transport and chemical processes other than the epoxide pathway^35^ to MTLs and MGA concentrations are small during this period. As shown in Figures S11 and S12, regional transport only affected epoxide concentrations by 5–13% in summer, and even lower during daytime. While during winter, regional transport generally had significant impacts on epoxide abundances by lowering their concentrations up to 21–96%. This ensures that the changes in total MTLs and MGA concentrations are mainly determined by formation through the acid-driven heterogeneous processes and removal by gas phase OH radicals, as illustrated in Figure 1. A more detailed explanation is provided in Text S3. Since MTLs and MGA are semivolatile compounds, we further consider their gas-aqueous–organic partitioning. The total mass variations of MTLs and MGA can be expressed by eqs 1 and 2, respectively

1 $$\frac{\Delta \left[{MTLs}_{tot}\right]}{\Delta t}=\left(1-\beta_{MTL‐OS}\right)k_{het_{IEPOX}}\left[IEPOX\right]-k_{OH+MTLs}\left[{OH}_{gas}\right]\left[{MTLs}_{gas}\right]$$

2 $$\frac{\Delta \left[{MGA}_{tot}\right]}{\Delta t}=\left(1-\beta_{MGA‐OS}\right)k_{het_{HMML/MAE}}\left[HMML+MAE\right]-k_{OH+MGA}\left[{OH}_{gas}\right]\left[{MGA}_{gas}\right]$$

where Δ*t* is the time duration of each measurement (7200 s). [IEPOX], [HMML], and [MAE] are molar concentrations of epoxides in the gas phase derived from OBM. $k_{het_{IEPOX}}$ and $k_{het_{HMML/MAE}}$ are the pseudo first-order heterogeneous reaction rate coefficients to be determined for IEPOX and HMML/MAE, respectively. Here we assume that the heterogeneous reaction rate coefficients of HMML and MAE are equal. However, it is worth noting that the aqueous phase reaction rate of HMML is probably faster than that of MAE under ambient conditions according to previous publications.^36,37^ [OH_gas_] is the gas-phase concentration of OH radicals which is also obtained from OBM. *k*_OH+MTLs_ and *k*_OH+MGA_ are the second-order reaction rate coefficients of MTLs (3.66 × 10^–11^ cm^3^ mol^–1^ s^–1^) and MGA (6.87 × 10^–12^ cm^3^ mol^–1^ s^–1^) with gas-phase OH radicals, respectively.^33^ [MTL_gas_] and [MGA_gas_] are gas-phase molar concentrations of MTLs and MGA, respectively. [MTLs_tot_] and [MGA_tot_] are total molar concentrations of MTLs and MGA, which include concentrations in the aqueous, organic, and gas phases. The impacts of background air entrainment due to PBL height growth on *k*_het_ estimations are narrowed by selecting afternoon data from 12:00 to 16:00 (local time). More detailed discussions can be referred to Text S5.

**Figure 1:**
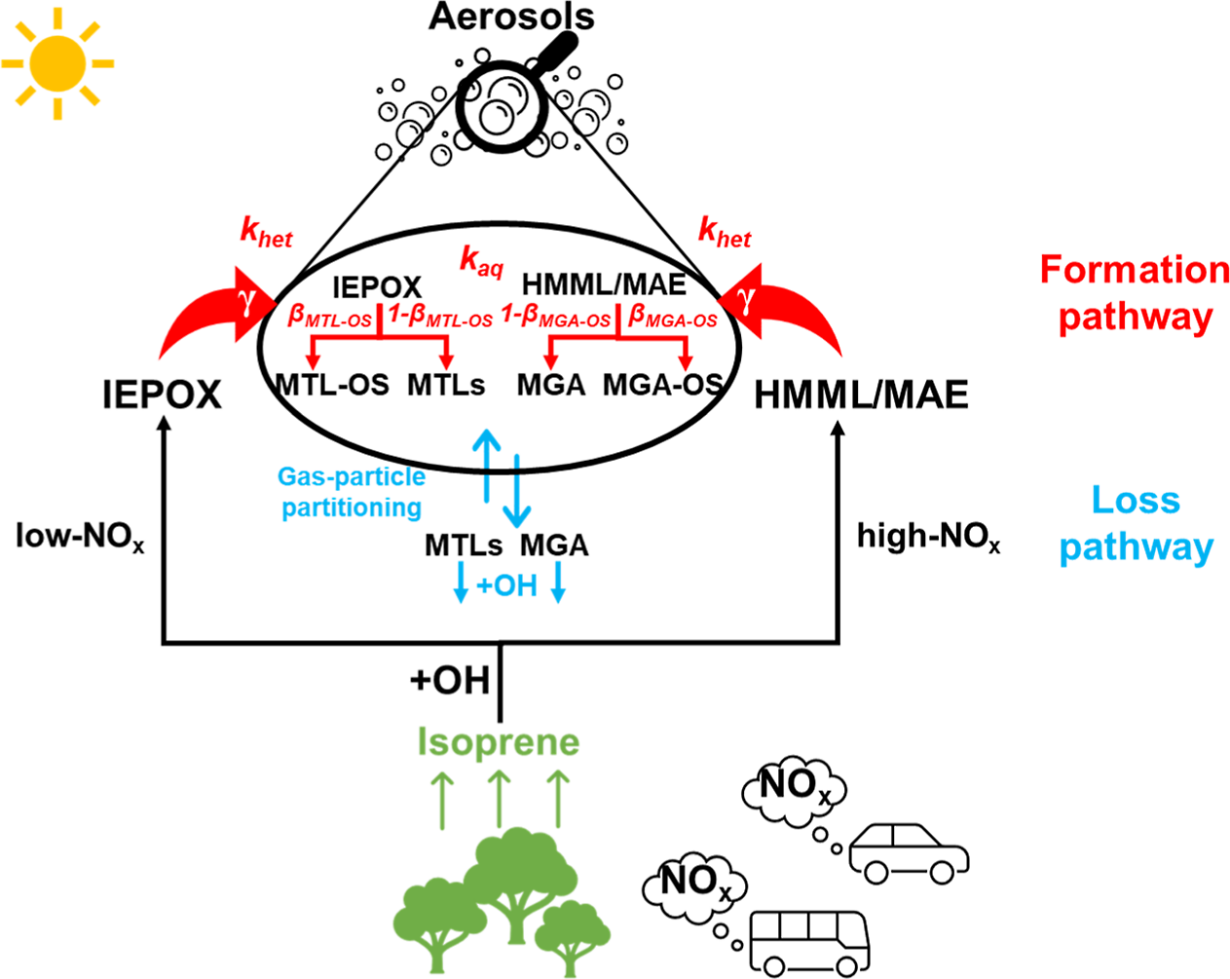
Schematic of the major daytime formation and loss pathways of 2-methyltetrols (MTLs) and 2-methylglyceric acid (MGA).

In this study, MTLs and MGA only in the particle phase were measured, representing the sum of concentrations in the aqueous and organic phases. The equilibrium concentrations in the aqueous, organic, and gas phases are estimated using the absorptive gas-organic partitioning theory^38^ and the gas-aqueous partitioning based on Henry’s Law. Details of the calculation are given in Text S4.

In addition to MTLs and MGA, organosulfates also account for an important fraction of SOA products from the epoxide pathway.^5,39–42^ In eqs 1 and 2, the branching ratio parameters β_MTL‑OS_ and β_MGA‑OS_ are used to indicate the fraction of reacted epoxides that form their respective organosulfates, and the terms (1 – β_MTL‑OS_) and (1 – β_MGA‑OS_) represent the branching ratios of the MTLs and MGA formation pathways, respectively. As online measurements of organosulfates were not available during this campaign. The values of β_MTL‑OS_ and β_MGA‑OS_ are determined by an iterative solution approach (Text S6).

The aqueous phase removals of MTLs and MGA by OH radicals and their deposition losses are not included in the eqs 1 and 2. The sensitivity analysis in Text S7 shows that the inclusion of aqueous phase removals by OH radicals, using the estimated aqueous OH radical concentrations based on the method of Zhang et al.,^33^ resulted in negligible changes in *k*_het_ (or γ) for MGA and a 25% increase in *k*_het_ (or γ) for MTLs during the campaign compared to the gas-phase removal pathway only. Besides, the sensitivity analysis in Text S8 shows that excluding deposition removal process resulted in a less than 33% decrease in estimated *k*_het_ for both IEPOX and HMML/MAE. Considering the larger uncertainties in the estimation of aqueous OH concentrations and deposition velocities, this omission would not significantly affect the overall results of this study.

After obtaining bihourly values of *k*_het_, the γ values of epoxides can be further calculated by eq 3 ^7^

3 $$\gamma =\frac{4k_{het}}{\omega S_{a}},,\omega =\sqrt{\frac{8RT}{\pi M}}$$

where *S*_a_ (m^2^ m^–3^) is the surface area of the aerosol particles obtained from the particle number size distribution measured by SMPS and APS. ω is the mean molecular velocity of the corresponding epoxide (m s^–1^). *M* (g mol^–1^) is the molecular weight of the corresponding epoxide, and *R* is the universal gas constant (J mol^–1^ K^–1^).

### 2.4 Estimations of Aqueous-phase Reaction Constants for Isoprene Epoxides

After determining γ using the method described in Section 2.3, the multiplication of the rate constant (*k*_aq_, s^–1^) for the aqueous phase reaction and the Henry’s Law coefficient (*H*_aq_, M atm^–1^) of the precursor epoxides can be calculated from eq 4 using the Γ_aq_ value determined from eq 5 ^7^

4 $$k_{aq}H_{aq}=\frac{\omega S_{a}\Gamma_{aq}}{4VRT}$$

5 $$\frac{1}{\Gamma_{aq}}=\frac{1}{\gamma }-\frac{R_{p}\omega }{4D_{g}}-\frac{1}{\alpha }$$

where *R*_p_ is the median particle radius (m) based on particle surface area distribution, and *V* is the total particle volume concentration (m^3^ m^–3^). *R*_p_ and *V* are both obtained from SMPS and APS measurements of the particle number size distribution. *D*_g_ is the gas phase diffusion coefficient, which is assumed to be 1 × 10^–5^ m^2^ s^–1^ for all isoprene epoxides.^7^ α is the mass accommodation coefficient and a value of 0.1 is adopted for all epoxides^7^ in this study. We calculate *k*_aq_ *H*_aq_ (M atm^–1^ s^–1^), instead of *k*_aq_, based on *H*_aq_ from the literature because the reported *H*_aq_ for epoxides in various model calculations and laboratory measurements can span several orders of magnitude,^7,8,10,43–45^ and the product *k*_aq_ *H*_aq_ can be treated as a single term when applying eqs 4 and 5 to determine the uptake coefficient γ in modeling studies.

Since the aqueous phase reaction rates of epoxides are largely dependent on particle acidity and nucleophilic addition,^7,8^ we further estimate their three reaction parameters, $H_{aq}k_{{H_{2}O,H}^{+}}$ , $H_{aq}k_{H_{2}O,{HSO}_{4}^{-}}$ and $H_{aq}k_{{{\text{SO}}_{4}^{2-},H}^{+}}$ , from eq 6 using multiple linear regression^46^ from a total of 109 bihourly values of *k*_aq_, *H*_aq_, [H^+^], [HSO_4_^–^] and [SO_4_^2–^]­

6 $${\left(k_{aq}H_{aq}\right)}_{i}=k_{{H_{2}O,H}^{+}}H_{aq}{\left(\left[H_{2}O\right]\left[H^{+}\right]\right)}_{i}+k_{{H_{2}O,HSO}_{4}^{-}}H_{aq}{\left(\left[H_{2}O\right]\left[{HSO}_{4}^{-}\right]\right)}_{i}+k_{{{\text{SO}}_{4}^{2-},H}^{+}}H_{aq}{\left(\left[{\text{SO}}_{4}^{2-}\right]\left[H^{+}\right]\right)}_{i},,i=1,2,...,109$$

where [H_2_O] is 55.6 mol L^–1^. The molar concentrations of H^+^ and HSO_4_^–^ are calculated using the widely used thermodynamic model ISORROPIA-II,^47–49^ with the MARGA-measured ionic composition data as inputs.

## 3 Results and Discussion

### 3.1 Overview of Field Campaign

Figure 2 shows the measured bihourly concentrations of isoprene and its early generation oxidation products (MACR + MVK) as well as later generation oxidation products in the particle phase (MTL_p_, and MGA_p_) at the SAES site during the summer (from July 1 to September 4). The gas phase concentrations of MTLs (MTL_g_) and MGA (MGA_g_) shown in Figure 2 are estimated from the measured particle phase concentrations and the gas-particle partitioning theory described in Text S4, and isoprene epoxides (IEPOX, HMML + MAE) are derived from OBM. Temporal variations of these species during the whole observation period are shown in Figure S10. On average, the measured concentrations of isoprene, MACR + MVK, MTL_p_ and MGA_p_ in summer were 0.43 ± 0.46 ppb, 0.75 ± 0.60 ppb, 3.5 ± 2.9 ng m^–3^, and 0.2 ± 0.3 ng m^–3^, respectively. These values were approximately 1–2 orders of magnitude higher than those observed in other months (Table S3, Figure S10), suggesting that biogenic SOA can play an important role in urban atmosphere during warm seasons.

**Figure 2:**
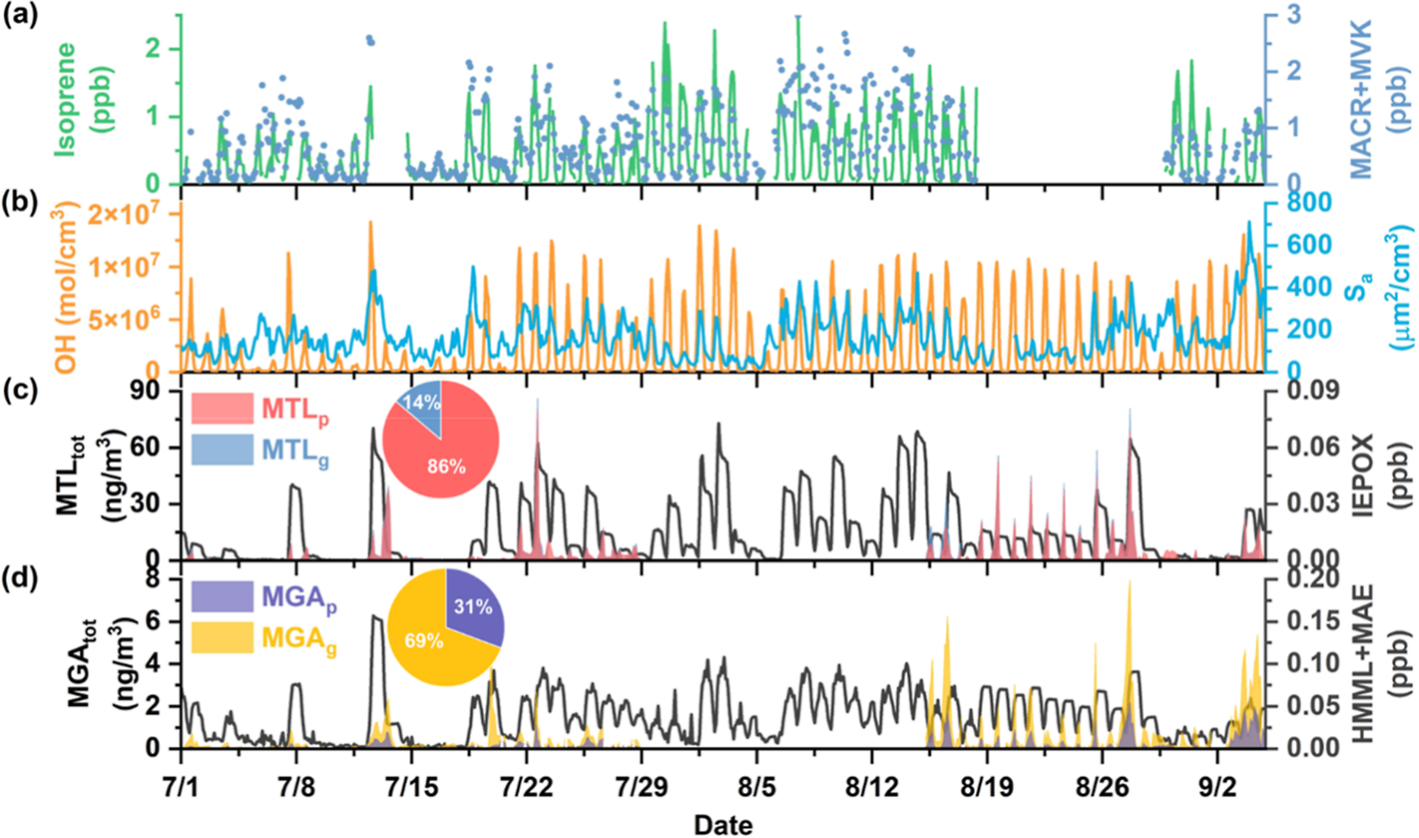
Summertime bihourly variations of (a) isoprene and its first-generation oxidation products (MACR + MVK) measured by GC-FID; (b) OH radicals simulated by OBM and particle surface area (*S*_a_) obtained from SMPS measurements; (c) particle-phase MTLs measured by the TAG system and their gas-phase concentrations estimated by gas-particle partitioning equilibrium theory, gas-phase precursor IEPOX (dark line) simulated by OBM; and (d) particle-phase MGA measured by the TAG system and its gas-phase concentrations estimated by gas-particle partitioning equilibrium theory, gas-phase precursor HMML + MAE (dark line) simulated by OBM. Their time series during the whole campaign are shown in Figure S10.

Similarly, gas-phase intermediates, IEPOX and HMML + MAE modeled by OBM also displayed significantly higher concentrations in summer compared with other seasons (Table S3, Figure S10). It is noteworthy that although the concentrations of HMML + MAE are more than 2 times higher than those of IEPOX, the abundance of their SOA product (MGA_tot_) was on average lower by a factor of 3 than that of MTLs (MTL_tot_) formed from IEPOX with nearly 70% of its mass partitioned into the gas phase (Figure 2).

Both MTLs and MGA showed similar temporal variations with their corresponding precursors IEPOX and HMML + MAE, especially during the summer daytime (Figure S22). Besides the abundances of precursors, higher concentrations of SOA products (e.g., MTLs and MGA) were also observed during periods characterized by higher levels of OH radicals (*R* > 0.35) and total particle surface area (*S*_a_) (*R* > 0.40), suggesting that OH oxidation and subsequent heterogeneous processes play an important role in isoprene SOA formation in the atmosphere. Consistent with previous chamber studies,^3–5^ NO_*x*_ is positively related to the formation of isoprene SOA products via either the IEPOX pathway or the HMML/MAE pathway, as indicated by the higher ratios of MGA to MTLs and HMML + MAE to IEPOX with increasing NO_*x*_ levels (*R* > 0.37) (Figure S23).

### 3.2 Estimations of *k*_het_ and γ

We next estimate the overall pseudo first-order rates for the heterogeneous reactions of isoprene epoxides in producing MTLs and MGA. In this study, the *k*_het_ of IEPOX related to real ambient conditions was estimated to be 5.5 × 10^–7^ to 3.4 × 10^–4^ s^–1^ during summer afternoon hours (12:00–16:00). The wide range of the estimated bihourly values of *k*_het_ is probably due to the changes in the chemical and physical properties of the particles, such as pH and surface area concentration. In comparison, the *k*_het_ of HMML/MAE was approximately 2 orders of magnitude lower than that of IEPOX, in the range of 1.2 × 10^–9^ to 5.3 × 10^–6^ s^–1^. Similarly, γ of IEPOX (5.1 × 10^–5^ to 2.6 × 10^–2^) was also about 2 orders of magnitude higher than that of HMML/MAE (5.6 × 10^–7^ to 4.2 × 10^–4^). It should be noted that the *k*_het_ and γ of HMML/MAE obtained here represent a combined effect of HMML and MAE uptake onto aerosols via heterogeneous reactions.

Figures 3 and S24 illustrate the effects of aerosol acidity ([H^+^]) and liquid water content (ALWC) on γ and *k*_het_ of isoprene epoxides, respectively. It is evident that higher values of *k*_het_ and γ were observed for both IEPOX and HMML/MAE when H^+^ had higher concentrations while ALWC had lower concentrations. Table 1 summarizes the estimated values of γ and *k*_het_ under different particle pH levels. The average value of γ for IEPOX increased from (2.1 ± 1.5) × 10^–3^ under particle pH > 4 to (6.7 ± 6.0) × 10^–3^ under pH < 2. For HMML/MAE, their γ values increased from (2.2 ± 1.9) × 10^–5^ under pH > 4 to (1.3 ± 1.1) × 10^–4^ under pH < 2. In other words, more acidic conditions promote the uptake of isoprene epoxides and facilitate the overall heterogeneous reactions. This result is consistent with conclusions from chamber experiments.^6,7,50^ Compared with previous studies, our estimates were generally comparable to chamber-derived values under conditions of similar particle pH and relative humidity (RH) (Table 1). However, they were generally 1–3 orders of magnitude higher than those from field studies^42,51,52^ predicted by equations using parameters (i.e., Henry’s Law constants, aqueous-phase reaction rate constants) obtained from laboratory studies. The reported values of these parameters span 1 to 2 orders of magnitude,^7,8,10,43–45^ resulting in large uncertainties in the estimates of γ and *k*_het_ (Section 3.3).

**Figure 3:**
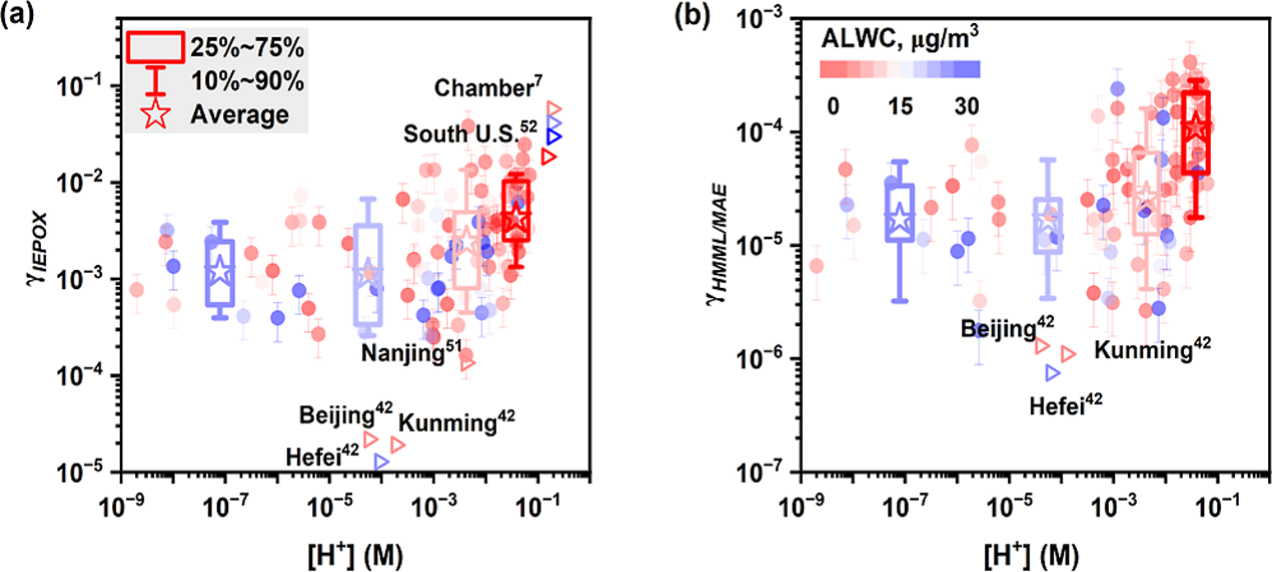
Reactive uptakes (γ) of (a) IEPOX and (b) HMML/MAE as a function of aerosol acidity ([H^+^]). The data points and boxes are colored by aerosol liquid water content (ALWC).

**Table 1:** Comparisons of Heterogeneous Reaction Rates (*k*_het_) and Uptake Coefficients (γ) of IEPOX and HMML/MAE Estimated in This Study with Previous Reported Values

	heterogeneous reaction rate coefficient (*k*_het_, s^–1^)		uptake coefficient (γ)		
reference	IEPOX	HMML/MAE	IEPOX	HMML/MAE	conditions
this study (Shanghai, China)	(7.3 ± 6.7) × 10^–5^	(1.4 ± 1.3) × 10^–6^	(6.7 ± 6.0) × 10^–3^	(1.3 ± 1.1) × 10^–4^	0 < pH ≤ 2 RH ∼ 58%
	(4.5 ± 3.5) × 10^–5^	(5.9 ± 5.3) × 10^–7^	(4.4 ± 3.3) × 10^–3^	(5.3 ± 5.2) × 10^–5^	2 < pH ≤ 3 RH ∼ 76%
	(2.7 ± 3.3) × 10^–5^	(3.1 ± 3.7) × 10^–7^	(2.6 ± 1.7) × 10^–3^	(2.9 ± 2.7) × 10^–5^	3 < pH ≤ 4 RH ∼ 76%
	(2.2 ± 1.3) × 10^–5^	(2.3 ± 1.9) × 10^–7^	(2.1 ± 1.5) × 10^–3^	(2.2 ± 1.9) × 10^–5^	pH > 4 RH ∼ 75%
Zhang et al., 2017 ^51^ (Nanjing, China)	7.1 × 10^–8^		1.6 × 10^–4^		pH ∼ 2.6 RH ∼ 60%
Zhang et al., 2022 ^42^ (Hefei, China)	5.9 × 10^–7^	1.7 × 10^–7^	1.3 × 10^–5^	7.5 × 10^–7^	pH ∼ 4.2 RH ∼ 73%
Zhang et al., 2022 ^42^ (Beijing, China)	8.3 × 10^–7^	3.1 × 10^–7^	2.3 × 10^–5^	1.3 × 10^–6^	pH ∼ 4.4 RH ∼ 49%
Zhang et al., 2022 ^42^ (Kunming, China)	7.5 × 10^–7^	1.4 × 10^–7^	2.0 × 10^–5^	1.1 × 10^–6^	pH ∼ 3.9 RH ∼ 68%
Xu et al., 2016 ^52^ (southeastern U.S.)	1.8 × 10^–3^		3.0 × 10^–2^		pH ∼ 1.2
Gaston et al., 2014 ^7^ (chamber)			(1.0 ± 0.1) × 10^–1^		NH_4_HSO_4_RH ∼ 30%
			(7.0 ± 3.0) × 10^–2^		NH_4_HSO_4_RH ∼ 50%
			(6.0 ± 1.0) × 10^–2^		NH_4_HSO_4_RH ∼ 70%
			(3.0 ± 0.8) × 10^–2^		pH ∼ 0.5 RH ∼ 50%
			(1.0 ± 0.5) × 10^–2^		pH ∼ 1 RH ∼ 50%
Riedel et al., 2015 ^6^ (chamber)			(6.5 ± 6.4) × 10^–4^		pH ∼ 4.1 RH ∼ 50%
			(9.4 ± 3.0) × 10^–3^		pH ∼ 0.14 RH ∼ 53%
			(1.1 ± 0.3) × 10^–2^		pH ∼ 1.4 RH ∼ 8%
				(4.9 ± 1.0) × 10^–4^	pH ∼ 0.14 RH ∼ 3%

### 3.3 Estimations of Aqueous-Phase Reaction Parameters

In this work, we report *k*_aq_ *H*_aq_ (M atm^–1^ s^–1^) instead of *k*_aq_ (s^–1^) values, as the product *k*_aq_ and *H*_aq_ can be treated as a single term when in calculating uptake coefficient γ. This also avoids dealing with the uncertainties associated with *H*_aq_ for IEPOX and MAE/HMML, which can vary by 1 to 2 orders of magnitude based on different model calculations and laboratory measurements.^7,8,10,43–45^ Figure 4 shows the estimated bihourly values of *k*_aq_ *H*_aq_ for IEPOX and HMML/MAE. *k*_aq_ *H*_aq_ for IEPOX ranges from 4.5 × 10^3^ to 3.7 × 10^6^ M atm^–1^ s^–1^ with an average value of (3.6 ± 5.4) × 10^5^ M atm^–1^ s^–1^. In comparison, *k*_aq_ *H*_aq_ values for HMML/MAE are approximately 2 orders of magnitude lower than those for IEPOX with an average of (5.3 ± 6.9) × 10^3^ M atm^–1^ s^–1^. In general, higher *k*_aq_ *H*_aq_ values are observed when the particles are more acidic and the ALWC is lower for both IEPOX and HMML/MAE.

**Figure 4:**
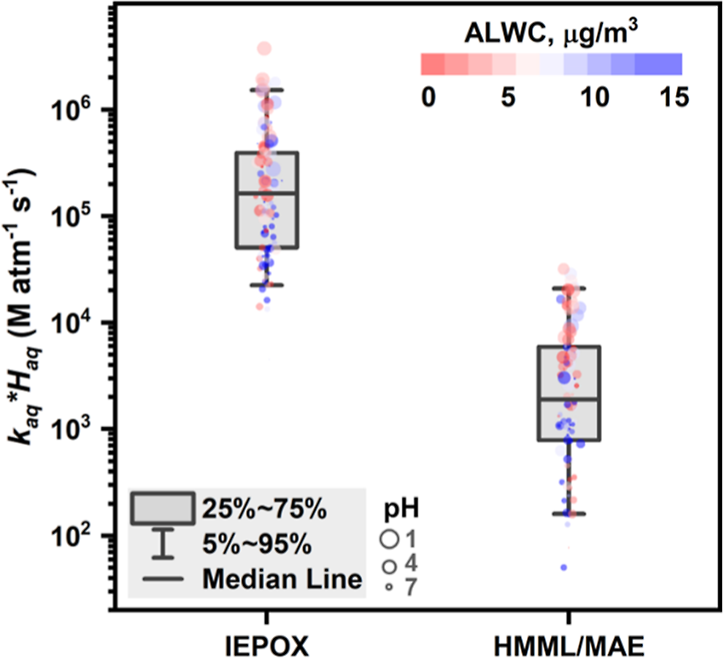
Estimated bihourly values of *k*_aq_ *H*_aq_ for IEPOX and HMML. The data points are colored by ALWC and sized by aerosol acidity (pH).

As shown in Table 2, the three parameters of aqueous reactions ( $k_{{H_{2}O,H}^{+}}H_{aq}$ , $k_{H_{2}O,{HSO}_{4}^{-}}H_{aq}$ and $k_{{{\text{SO}}_{4}^{2-},H}^{+}}H_{aq}$ ) for IEPOX and HMML/MAE were further determined with eq 6 using multivariate linear regression (MLR) analysis.^46^ Uncertainties in the MLR results were investigated using the bootstrap technique. Details of the bootstrap analysis results are provided in Table S6. Uncertainties brought by [H^+^] and ALWC on the parameter estimations are presented in Table S7. Previous bulk solution experiments have reported $k_{{H_{2}O,H}^{+}}$ and $k_{{{\text{SO}}_{4}^{2-},H}^{+}}$ for IEPOX to be (1.4–9.0) × 10^–4^ M^–2^ s^–1^ and (2.0–4.8) × 10^–4^ M^–2^ s^–1^, resulting in $k_{{H_{2}O,H}^{+}}H_{aq}$ and $k_{{{\text{SO}}_{4}^{2-},H}^{+}}H_{aq}$ ranging from 2.7 × 10^3^ to 8.6 × 10^5^ and from 3.8 × 10^3^ to 4.6 × 10^5^ M^–1^ atm^–1^ s^–1^, respectively, using the ranges of *H*_aq_ values reported in the literature. The estimated values of $k_{{H_{2}O,H}^{+}}H_{aq}$ , $k_{H_{2}O,{HSO}_{4}^{-}}H_{aq}$ and $k_{{{\text{SO}}_{4}^{2-},H}^{+}}H_{aq}$ for IEPOX in our study are (5.9 ± 1.3) × 10^4^, (2.1 ± 0.3) × 10^5^, and (6.7 ± 1.3) × 10^4^ M^–1^ atm^–1^ s^–1^, respectively (Table 2), which are close to the upper limit of this range. This indicates that higher *H*_aq_ values (e.g., 6.5 × 10^7^ to 4.2 × 10^8^ M atm^–1^) may be required when predicting *k*_het_ and γ with the parametrization method.

**Table 2:** Comparisons of Acid-Catalyzed Aqueous-Phase Reaction Constants for IEPOX and HMML/MAE Estimated in This Study with Previous Reported Values^a^

	IEPOX (M^–1^ atm^–1^ s^–1^)			HMML/MAE (M^–1^ atm^–1^ s^–1^)		
reference	$k_{{H_{2}O,H}^{+}}H_{aq}$	$k_{H_{2}O,{HSO}_{4}^{-}}H_{aq}$	$k_{{{\text{SO}}_{4}^{2-},H}^{+}}H_{aq}$	$k_{{H_{2}O,H}^{+}}H_{aq}$	$k_{H_{2}O,{HSO}_{4}^{-}}H_{aq}$	$k_{{{\text{SO}}_{4}^{2-},H}^{+}}H_{aq}$
This study	(5.9 ± 1.3) × 10^4^	(2.1 ± 0.3) × 10^5^	(6.7 ± 1.3) × 10^4^	(1.5 ± 0.8) × 10^3^	(3.4 ± 0.7) × 10^3^	(7.3 ± 2.7) × 10^2^
Birdsall et al., 2014^b^ ^36^	2.7 × 10^3^–1.3 × 10^5^			1.3–8.3		
Riedel et al., 2016^b^ ^12^	6.5 × 10^3^–3.3 × 10^5^		9.1 × 10^3^–4.6 × 10^5^			
Pye et al., 2013^b^ ^10^	1.7 × 10^4^–8.6 × 10^5^	2.5 × 10^2^–1.2 × 10^3^	3.8 × 10^3^–1.9 × 10^5^			
Piletic et al., 2013^c^ ^11^	1.0 × 10^6^–5.1 × 10^7^		9.9 × 10^6^–5.0 × 10^8^	36–225		

a The standard deviations of the estimated values in this study are derived from the bootstrap analysis (Table S6).

b Ranges were obtained by multiplying $k_{{H_{2}O,H}^{+}}$ , $k_{H_{2}O,{HSO}_{4}^{-}}$ , and $k_{{{\text{SO}}_{4}^{2-},H}^{+}}$ values from bulk solution-phase experiments with reported upper (9.6 × 10^8^ M atm^–1^ for IEPOX and 7.5 × 10^6^ M atm^–1^ for HMML/MAE) and lower (1.9 × 10^7^ M atm^–1^ for IEPOX and 1.2 × 10^6^ M atm^–1^ for HMML/MAE) values of *H*_aq_.

c Ranges were obtained by multiplying $k_{{H_{2}O,H}^{+}}$ , $k_{H_{2}O,{HSO}_{4}^{-}}$ , and $k_{{{\text{SO}}_{4}^{2-},H}^{+}}$ values from density functional theory and transition state theory with reported upper and lower values of *H*_aq_.

A few studies have reported the $k_{H_{2}O,{HSO}_{4}^{-}}$ value for IEPOX. Pye et al.^10^ used 1.3 × 10^–5^ M^–2^ s^–1^ based on the estimated value for *cis*-2,3-epoxybutane-1,4-diol by Eddingsaas et al.,^8^ which is approximately an order of magnitude lower than $k_{{H_{2}O,H}^{+}}$ and $k_{{{\text{SO}}_{4}^{2-},H}^{+}}$ values used in their study. In our estimates, the values predicted for HMML/MAE were generally 2 orders of magnitude lower than those for IEPOX in our study. Using a $k_{{H_{2}O,H}^{+}}$ value of 1.1 × 10^–6^ M^–2^ s^–1^ for MAE,^36^ the *H*_aq_ for HMML/MAE would reach to 1.4 × 10^9^ M atm^–1^, which is much higher than previously estimated values (1.2 × 10^6^ to 7.5 × 10^6^ M atm^–1^ for MAE) based on chemical structures. Jiang et al. (2018)^37^ chose to study β-propiolactone as a proxy system for the reactivity of HMML and found that the aqueous-phase reaction rate of HMML may be significantly higher than that of MAE under ambient conditions, since HMML undergoes a general acid-catalyzed process^37^ while MAE is found to undergo a Bronsted acid-catalyzed process.^36^ Considering that the average concentration of HMML was predicted to be around 3 times higher than that of MAE during the campaign, it is possible that the significant higher value of $k_{{H_{2}O,H}^{+}}H_{aq}$ obtained in this study is due to the higher aqueous reaction rates of HMML. Thus, parameters obtained from bulk experiments for MAE may not represent HMML well, which in turn leads to large uncertainties in estimating isoprene SOA formation under high NO_*x*_ conditions. Using filter-based measurements, Zhang et al.^42^ also found that current parameters^10–12^ of aqueous-phase reactions for HMML/MAE could cause significant underestimation of related SOA products. In other words, higher values of *H*_aq_ and/or reactive rate coefficients are needed to improve the agreement between model predictions and observations. Thus, these findings suggest that large uncertainties may exist in the fundamental data for partitioning parameters of MAE/HMML and reaction kinetics measured with authentic standard for HMML are needed to better describe the formation of isoprene SOA under high NO_*x*_ condition.

## 4 Implications

By taking advantage of high time-resolution measurements of organic molecular markers, this study provides a new approach to quantify real-world heterogeneous reaction rate coefficients (*k*_het_) and uptake coefficients (γ) of isoprene epoxides during summer daytime. Our estimates suggest that the isoprene SOA formation rate via the high NO_*x*_ pathway is significantly lower than that via the low NO_*x*_ pathway, as indicated by the 2–3 orders of magnitude lower values of *k*_het_ and γ for HMML/MAE than those for IEPOX. However, other high NO_*x*_ pathway processes, which contribute to the formation of isoprene SOA products other than MGA and organosulfates (e.g., organic nitrates), cannot be excluded. Additionally, results presented in this study reveal that model parameters, such as the Henry’s law constant (*H*_aq_), have crucial impacts on aqueous-phase reaction rate (*k*_aq_) calculations. By applying different values of *H*_aq_, the estimated *k*_aq_ for isoprene epoxides can vary by 2 orders of magnitude. Using aqueous-phase reaction constants (e.g., $k_{{H_{2}O,H}^{+}}$ ) of MAE to calculate *k*_aq_ for HMML may also lead to significant underestimation of overall heterogeneous reaction rates of isoprene epoxides via high NO_*x*_ pathway. Thus, the parametrization method, which greatly relies on *H*_aq_ and $k_{{H_{2}O,H}^{+}}$ to calculate *k*_aq_, has large uncertainties in predicting *k*_het_ and γ for isoprene epoxides. Therefore, it is necessary to measure kinetic parameters for authentic isoprene epoxides under atmospherically relevant conditions to further reduce uncertainties in the assessment of isoprene SOA on regional and global air quality and climate.

## Supplementary Material



## Notes

Details on measurement data quantification and quality control; model performance evaluation; data selected for estimations; estimation of aqueous-, organic-, and gas-phase concentrations of MTLs and MGA; estimation of the organosulfate formation branching ratios; and sensitivity analysis of impacts of planetary boundary layer, aqueous-phase loss pathway, deposition loss pathway on *k*_het_ estimations (PDF)

## ¶

Lawrence Berkeley National Laboratory, Berkeley, California 94720, United States

## ∇

Division of Environment and Sustainability, Hong Kong University of Science and Technology, Hong Kong, China.
