# Supplementary material for: Aerosol Uptake Coefficients of Isoprene Epoxides: Determination and Parameter Estimation from Online Field Measurements of Organic Molecular Markers
Source: Environ Sci Technol. 2025 Aug 22;59(34):18249–58. doi: 10.1021/acs.est.5c09046 (PMC12409889; doi:10.1021/acs.est.5c09046)
Supplement: Supplementary file 1 [file es5c09046_si_001.pdf]

Supporting Information for

Uptake coefficients and aqueous-phase reaction rate constants of isoprene epoxides: Estimates based on online measurements of organic molecular markers

Shuhui Zhu[1,2], Jie Zhang[3,‡], Li Li[4], Min Zhou[1], Liping Qiao[1], Hongli Wang[1], Dan Dan Huang[1], Qiongqiong Wang[5], Shengao Jing[1], Yuhang Wu[1], Shan Wang[2], Changhong Chen[1], Qi Ying[3,†,*], Jian Zhen Yu[2,6,*]

[1]State Environmental Protection Key Laboratory of Formation and Prevention of Urban Air Pollution Complex, Shanghai Academy of Environmental Sciences, Shanghai 200233, China

[2]Division of Environment and Sustainability, Hong Kong University of Science and Technology, Kowloon 999077, China

[3]Zachary Department of Civil and Environmental Engineering, Texas A&M University, College Station, Texas 77843-3136, United States

[4]Rural Environment Protection Engineering & Technology Center of Sichuan Province, College of Environmental Sciences, Sichuan Agricultural University, Chengdu 611830, China

[5] Department of Atmospheric Science, School of Environmental Studies, China University of Geosciences, Wuhan 430074, China

[6]Department of Chemistry, Hong Kong University of Science and Technology, Kowloon 999077, China

[‡]Now at Lawrence Berkeley National Laboratory, Berkeley, California 94720, United States

[†]Now at Division of Environment and Sustainability, Hong Kong University of Science and Technology, Hong Kong, China

[*]Corresponding author: Jian Zhen Yu (jian.yu@ust.hk) and Qi Ying (qying@ust.hk)

Contents of this file:

Text S1 to S8

Table S1 to S7

Figures S1 to S24

References

Text S1. Data quantification and quality control

A total of 560 bi-hourly aerosol samples were collected and measured by the TAG system throughout the field campaign. With the application of internal standard and external standard mixtures, a total of 102 organic molecules were identified and quantified in this study. Detail explanation of chemical analysis of other organic molecules can be referred to previous studies.[1-3] δ1-IEPOX was synthesized via hydrolysis of 2-methyl-2-vinyloxirane and then epoxidation of 3-methyl-1-butene-3,4-diol, following the synthesis procedure described by Li et al. (2023)[4]. 2-methylthreitol and 2-methylerythritol (MTLs) were identified and quantified with authentic standards synthesized[4]. To be specific, we found that a minute amount of 2-methylthreitol and 2-methylerythritol were also produced during the synthesis process of δ1-IEPOX. The peak area ratios of 2-methylthreitol and 2-methylerythritol to δ1-IEPOX was 0.11 and 0.18 (Figure S1), respectively. As these ratios remained almost unchanged in the later calibration solutions, the 2-methylthreitol and 2-methylerythritol present in the synthesized δ1-IEPOX can be used as standards to build calibration curves. For the establishment of calibration curves, we first dissolved the synthesized δ1-IEPOX in menthol and diluted it to 0.5 ppm (0.5 ng μL^{-1}). The concentrations of 2-methylthreitol and 2-methylerythritol in the diluted standard were 0.055 and 0.090 ng μL^{-1}, respectively. Then six different volumes (0, 5, 10, 15, 20, 25 μL) of the above diluted solution were mixed with a fixed volume (5 μL) of internal standard mixtures. The resulting solutions were injected into the TAG system and analyzed using the same GC/MS program as that for the samples. The calibration curves for MTLs (Figures S2) were established using succinic acid-d4 as the internal standard. During the ambient measurements, 5 μL of internal standard mixture was injected into each aerosol sample by the auto-injection system equipped in the TAG. The target organic compounds were identified by their retention times (Figure S3) and mass spectra (Figure S4), and their masses were quantified using the above established calibration curves. Since we do not have authentic standard for MGA, it is quantified with the calibration curve built for MTLs. Considering that MGA shares similar chemical structure with MTLs, the quantification uncertainties of MGA were expected to be small.

As part of quality control/quality assurance, blank samples and standard mixture samples were collected once a week. A total of 7 blank samples and 7 standard mixture samples were collected during the operation of the TAG system. The blank samples and standard mixture samples were analyzed using the same GC/MS program as those for the samples. As shown in Figure S5, the mass concentrations of IEPOX, 2-methylthreitol, 2-methylerythritol, and MGA in the blank samples were generally one to two orders of magnitude lower than those in aerosol samples. In other words, the presences of IEPOX, 2-methylthreitol, 2-methylerythritol, and MGA in the blank samples were all lower than 10% of the average concentration level detected in the samples. Thus, the TAG-measured concentrations of MTLs and MGA were not blank-corrected. The detection limits of the TAG system for IEPOX, 2-methylthreitol, 2-methylerythritol, and MGA were determined as their average concentrations in blank samples, which were 0.01, 0.06, 0.26, and 0.01 ng/m^3, respectively.

The error uncertainties in TAG sample measurements (E_{TAG}) were estimated by the following equation[5,6]:

$$\%E_{TAG} = \sqrt{(\%E_{FB})^2 + (\%E_R)^2 + (\%E_Q)^2} \qquad \text{S1}$$

$$\%E_Q = \%E_n \Delta n + \%E_f \Delta f + \%E_d \Delta d \qquad \text{S2}$$

where E_{FB} is relative error introduced by field blank, E_R is relative error introduced by spike recovery, and E_Q is error from surrogate quantification. Considering that many organic compounds normally show zero concentrations in blank samples with GC-MS detection technique, we calculated E_{FB} values based on the repeated concentrations of the lowest calibration standard, which are 1.9, 1.3, and 1.2 ng/m^3 for IEPOX, 2-methylthreitol, and 2-methylerythritol, respectively. The E_R values were determined with concentrations measured in standard mixture samples. For IEPOX and MTLs, their concentrations were quantified with authentic standards. Thus, their E_Q values equal to zero. For MAG, its E_Q was estimated via an empirical approach[5] with equation S2, where the relative error introduced by each carbon atom (E_n), oxygenated functional group (E_f) and alkenes (E_d) was estimated to be 15%, 10%, and 60%, respectively. Δn, Δf, and Δd are the differences in the number of carbon atom, oxygen-containing functional group, and alkene functionality between a surrogate and an analyte, respectively. In this study, E_{TAG} for IEPOX, 2-methylthreitol, 2-methylerythritol, and MGA were 4.0%, 7.0%, 8.6% and 15.0%, respectively (Table S1).

We further evaluated the quality of bi-hourly dataset by conducting multiple cross-comparisons among independent measurements, of which scatter correlation plots are shown in Figure S6. For example, the summed mass of 102 TAG-measured organic molecules is well correlated with OC measured by OC/EC analyzer (R = 0.86). TAG-measured MTLs and MGA show moderate correlations with isoprene (R = 0.38) and MACR+MVK (R = 0.25) measured by GC-FID, suggesting the importance of chemical processes on the abundances of isoprene SOA tracers. To validate the ion measurements, the anion and cation balance as well as the calculated versus measured ammonium (NH_4^+) in $PM_{2.5}$ are also compared. The concentrations of anions in μeq/m^3 are calculated by the equation ([Cl^-]/35.5+[NO_3^-]/62.0+[SO_4^{2-}]/48.0), and the concentrations of cations in μeq/m^3 are calculated by the equation ([NH_4^+]/18.0+[Na^+]/23.0+[K^+]/39.1). A strong correlation (R = 0.99) with a slope close to 1 is observed for the aerosol samples collected at the site. Since NH_4^+ is very often found in the chemical forms of NH_4NO_3, $(NH_4)_2SO_4$, and NH_4HSO_4, the concentrations of NH_4^+ can be calculated by assuming full neutralization. That is [NH_4^+] in μg/m^3 = 0.29 × [NO_3^-] + 0.38 × [SO_4^{2-}] based on NH_4NO_3 and $(NH_4)_2SO_4$, or [NH_4^+] in μg/m^3 = 0.29 × [NO_3^-] + 0.192 × [SO_4^{2-}] based on NH_4NO_3 and NH_4HSO_4. For both forms of sulfate, the comparisons show strong correlations with R > 0.98. Overall, the sum of measured species (Cl^-, NO_3^-, SO_4^{2-}, NH_4^+, Na^+, K^+, Mg^{2+}, Ca^{2+}, OC, EC) in $PM_{2.5}$ is well correlated with $PM_{2.5}$ measured by the particulate monitor (R = 0.86), indicating that the TAG system and other online instruments have provided good quality measurements during the campaign.

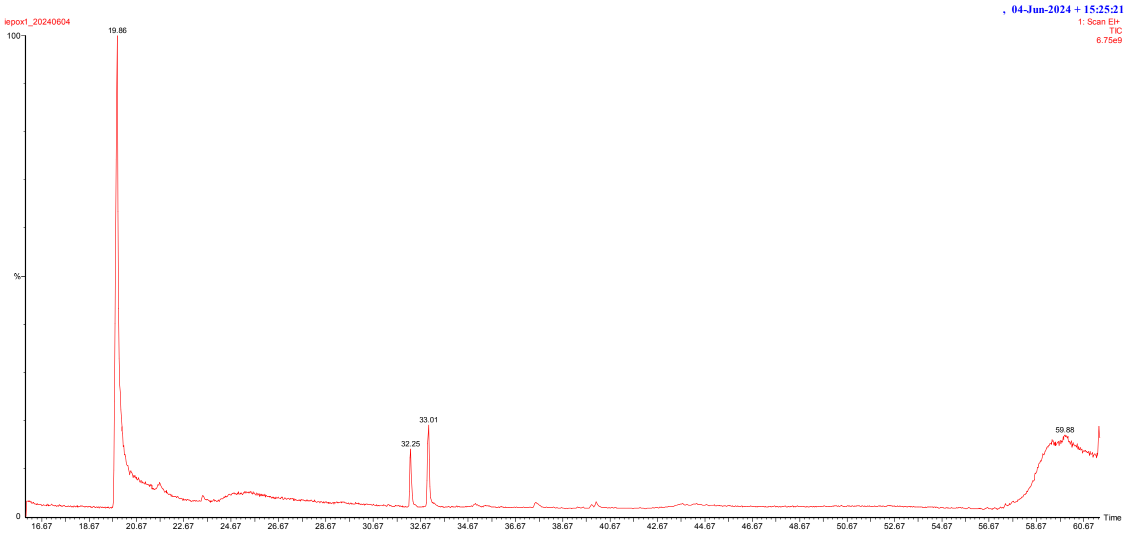

Figure S1. Peak areas of δ1-IEPOX, 2-methylthreitol, and 2-methylerythritol detected in the synthesized standard.

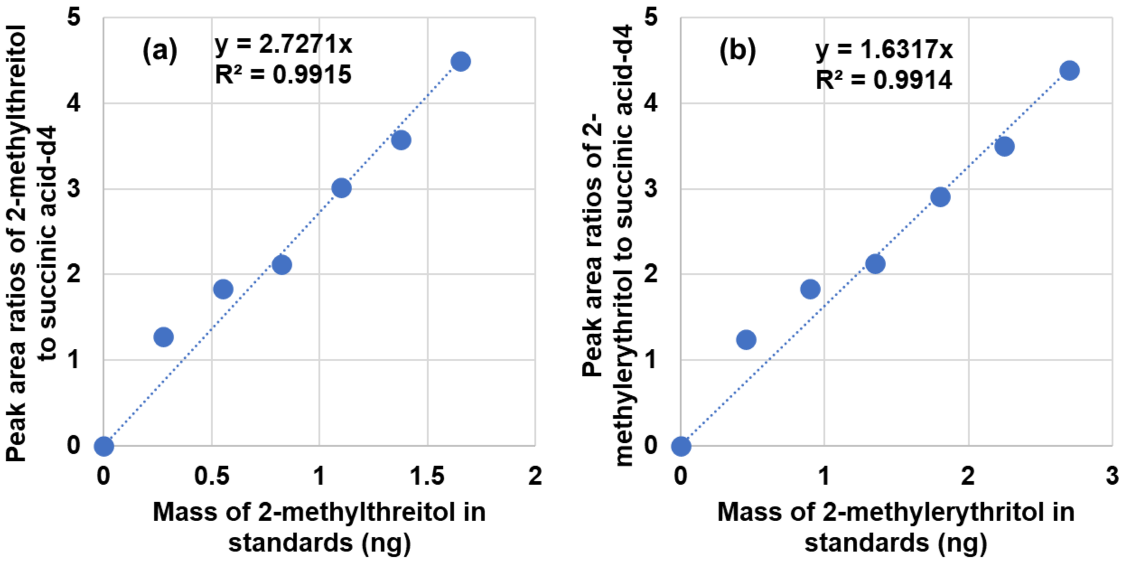

Figure S2. Calibration curves for (a) 2-methylthreitol and (b) 2-methylerythritol with authentic standard.

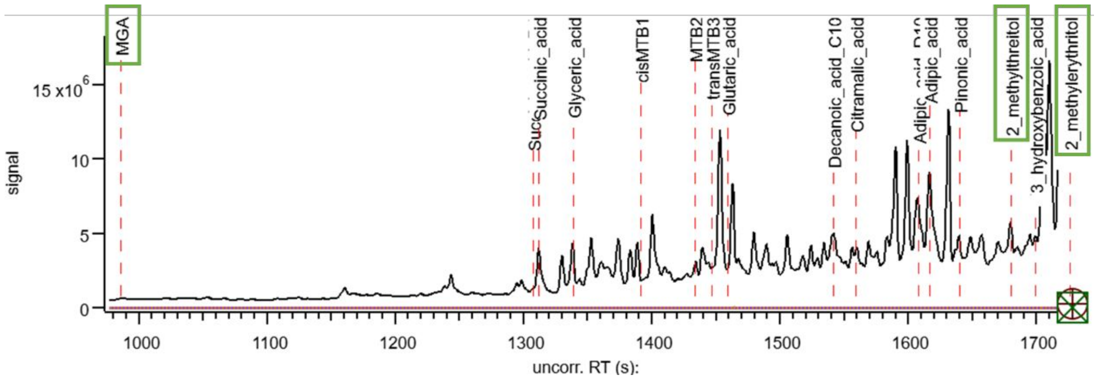

Figure S3. Total ion chromatogram (TIC) showing 2-methylglyceric acid (MGA), 2-methylthreitol, 2-methylerythritol, and other selected organic molecular markers.

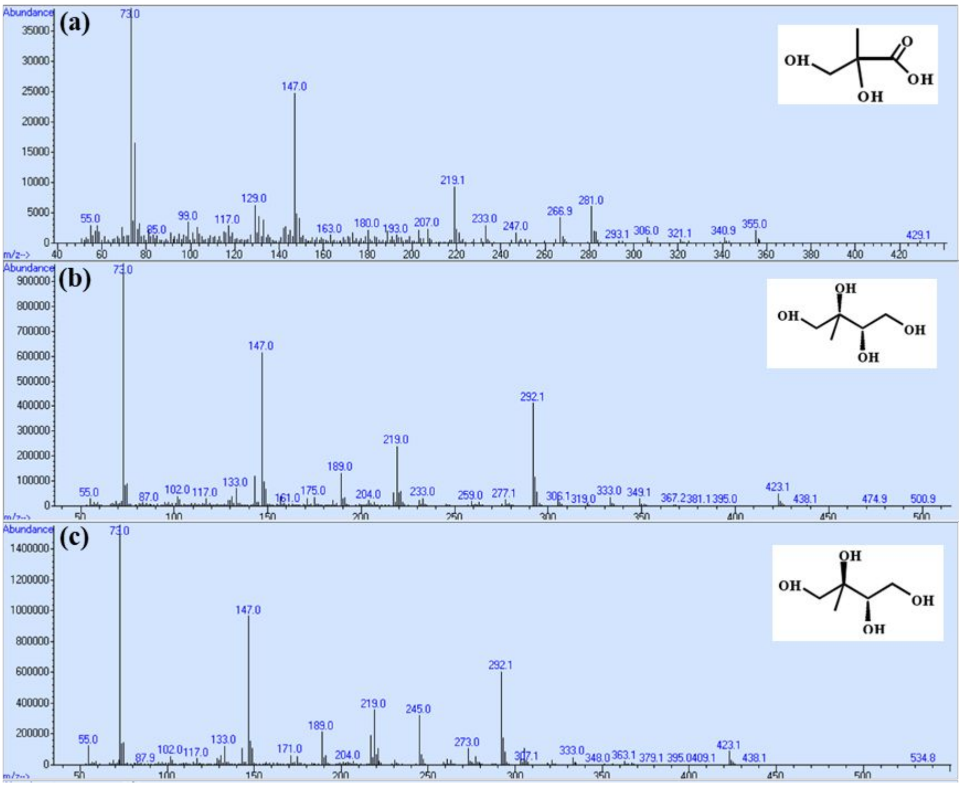

Figure S4. Mass spectra of the trimethylsilyl derivatives of (a) 2-methylglyceric acid (MGA), (b) 2-methylthreitol, and (c) 2-methylerythritol obtained from the TAG system.

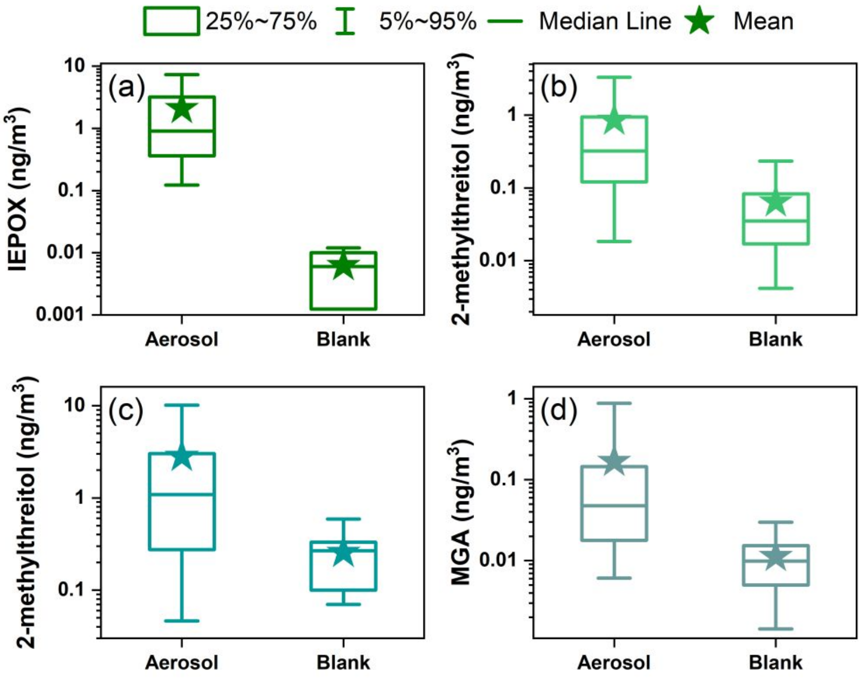

Figure S5. Mass concentrations of (a) IEPOX, (b) 2-methylthreitol, (c) 2-methylerythritol, and (d) 2-methylglyceric acid (MGA) in aerosol and blank samples.

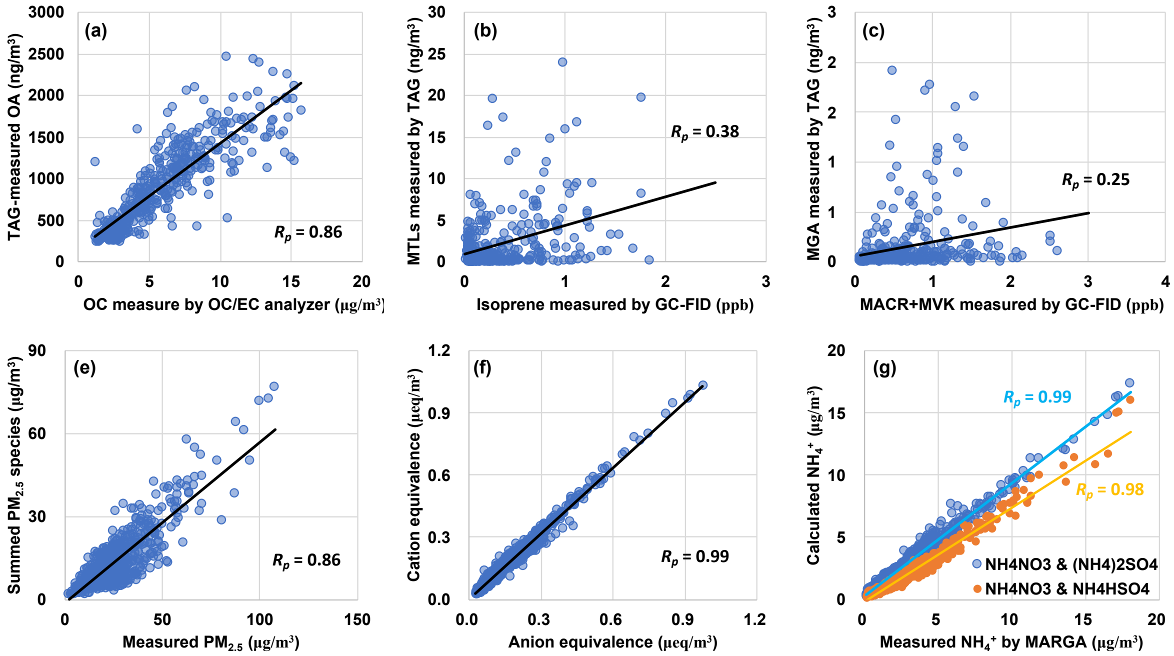

Figure S6. Scatter plots of select pairs of measured parameters with known underlying physical relationships. They serve as internal data consistency check.

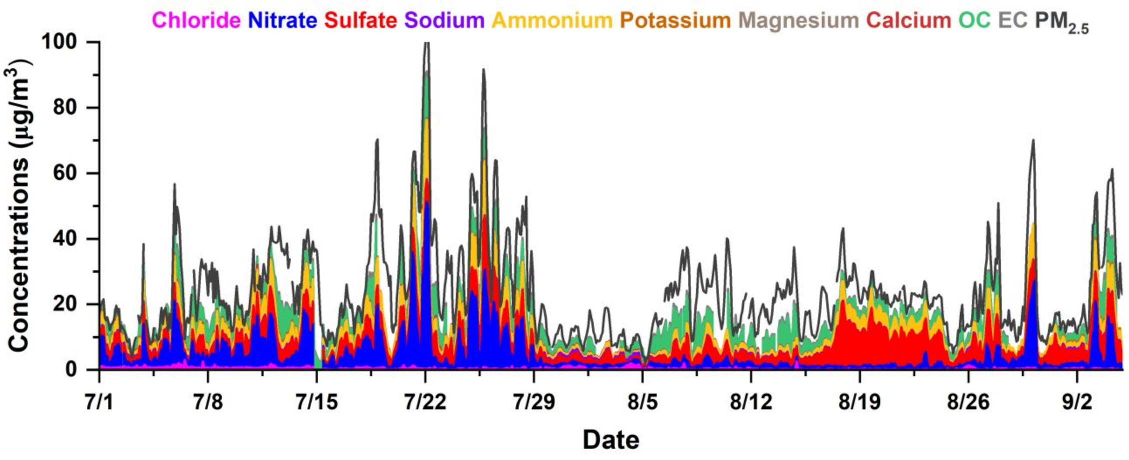

Figure S7. Time series of $PM_{2.5}$ masses and its major components during the investigation period.

Table S1. Uncertainties associated with TAG sample measurements.

compounds	E_{FB}	E_R	E_Q	E_{TAG}
IEPOX	1.1%	3.8%	0.0%	4.0%

2-methylthreitol	5.6%	4.2%	0.0%	7.0%
2-methylerythritol	6.7%	5.4%	0.0%	8.6%
MGA	0.0%	0.0%	15.0%	15.0%

Text S2. Evaluation of model performance

While methods for detection of IEPOX and HMML/MAE in ambient samples have been developed in several studies,[7-9] we find it is difficult to identify and quantify their gas-phase concentrations with our existing online measurement techniques at the site (e.g., PTR-ToF, CIMS). These difficulties arise due to their low abundances, strong reactivities, and possible co-presence of isobaric molecular formulas (e.g., 3-hydroxypentanoic acid). To evaluate the performance of the observation-based box model (OBM) adopted in this study, we compared concentrations derived from OBM with those measured by GC-FID for epoxide precursors (isoprene, MACR, MVK). The results confirm that chemical mechanisms included in the model generally succeeded in capturing the mass variations of isoprene and its oxidation products during the campaign (Figure S8).

An additional observation was conducted at the same site with authentic standard[4] of IEPOX in the summer of 2024, during which a calibration curve for IEPOX in particle-phase has been established for the TAG system. Using this calibration curve, we identified and re-quantified the mass concentrations of aerosol-phase IEPOX in the summer of 2020. Since the formation of MTLs in the particle phase can be expressed using the reactive uptake expression (left side of equation (S3)) as well as the aqueous reaction expression (right side of equation (S3)), thus the concentration of IEPOX in the particle-phase can be estimated with its gas-phase concentrations using equation (S4),

$$(1-\beta_{MTL-OS})k_{het_{IEPOX}}[IEPOX]_g = k_{aq}[IEPOX]_p \quad \text{(S3)}$$

$$[IEPOX]_p = \frac{(1-\beta_{MTL-OS})k_{het_{IEPOX}}[IEPOX]_g}{k_{aq}} \quad \text{(S4)}$$

where β_{MTL-OS} is the branching ratio of organosulfates formation pathway and is determined via method described in Text S6. k_{het} and k_{aq} are the overall heterogeneous reaction rates and aqueous-phase reaction rates of IEPOX, respectively. k_{het} (s^{-1}) is derived from this study via method stated in Section 3.2. k_{aq} (s^{-1}) is estimated from $k_{aq}H_{aq}$ (M atm^{-1} s^{-1}) obtained in Section 3.3 by selecting an appropriate value for H_{aq}. Figure S9 compares the particle-phase concentrations of IEPOX predicted by equations (S1) and (S2) with the TAG-measured concentrations using an H_{aq} value of 2.2×10^8 M atm^{-1}. Their strong correlations (R = 0.72) further support that model results can well represent isoprene oxidation in the real atmosphere. Besides, we also employed the bootstrap method to conduct sensitivity analysis of model results on the estimated coefficients. The bootstrap method allows us to assess the stability and robustness of our model estimates by generating multiple resampled datasets from the original data. Specifically, we repeatedly draw random samples with replacement from the OBM and TAG measurement dataset, each of which has the same sample size as the original dataset. For each resampled dataset, we derived the estimated coefficients through the same calculation procedure as stated in Method 2.3 and 2.4. The bootstrap results are given in Table S2. The mean values of bootstrap results with 1000 replications were in general close to the derived values listed in Table 2, suggesting that systematic errors brought by OBM in k_{het} and γ

estimations are likely limited.

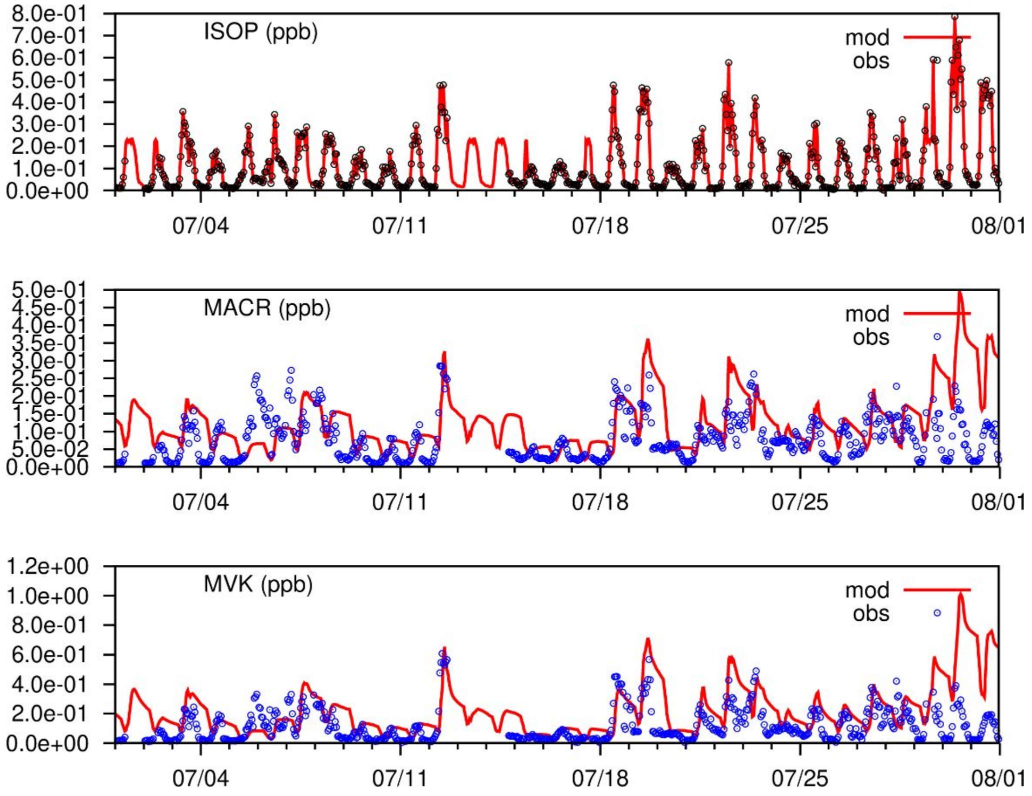

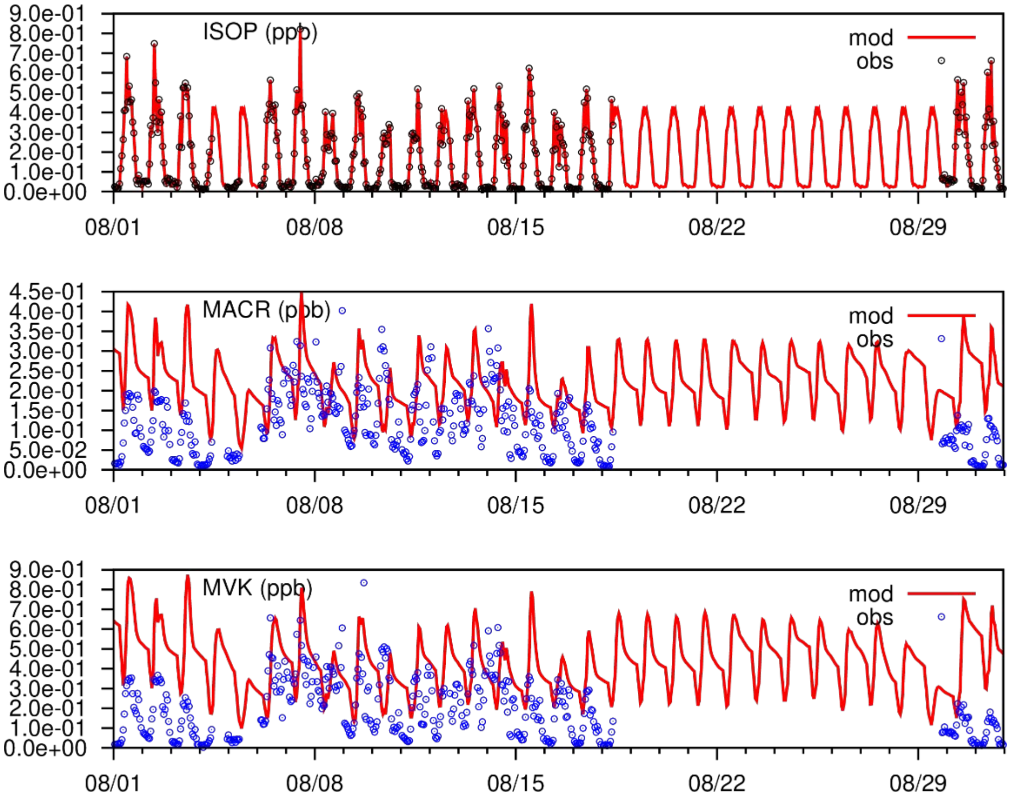

Figure S8. Time series of mass concentrations of isoprene, MACR, and MVK modeled by OBM (red lines) and measured by GC-FID (blue dots).

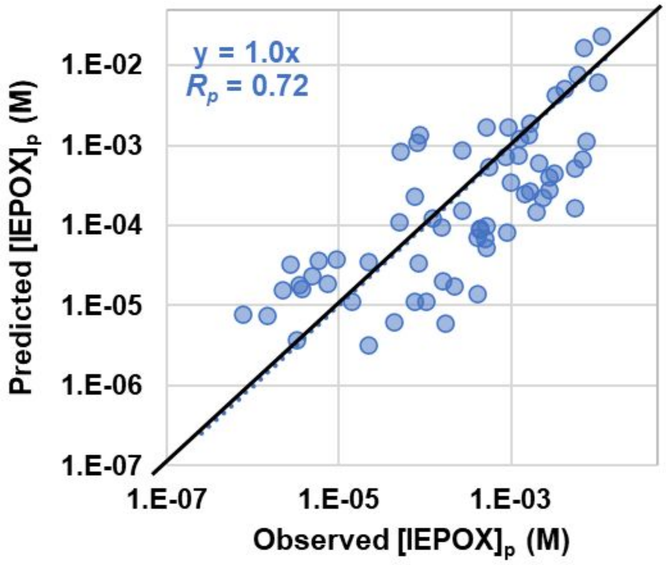

Figure S9. Scatter plot of particle-phase IEPOX concentrations predicted based on modeled gas-phase concentrations and its TAG-measured concentrations with a H_{aq} value of 2.2 × 10^8 M atm^{-1}.

Table S2. Sensitivity analysis of the model results on the estimated coefficients using bootstrap method.

Factors		mean	95% CI for mean	SD	95% CI for SD
IEPOX	k_{het}, s^{-1}	3.7 × 10^{-5}	[2.9 × 10^{-5}, 4.5 × 10^{-5}]	1.3 × 10^{-5}	[8.9 × 10^{-6}, 2.0 × 10^{-5}]
	β_{OS}	0.11	[0.10, 0.13]	0.06	[0.04, 0.08]
	$k_{H_2O,H^+}H_{aq}$, M^{-1} atm^{-1} s^{-1}	5.7 × 10^4	[3.1 × 10^4, 8.2 × 10^4]	2.2 × 10^4	[1.1 × 10^4, 3.2 × 10^4]
	$k_{H_2O,HSO_4^-}H_{aq}$, M^{-1} atm^{-1} s^{-1}	2.1 × 10^5	[1.3 × 10^5, 3.0 × 10^5]	8.5 × 10^4	[7.4 × 10^4, 9.5 × 10^4]
	$k_{SO_4^{2-},H^+}H_{aq}$, M^{-1} atm^{-1} s^{-1}	7.0 × 10^4	[4.7 × 10^4, 9.1 × 10^4]	2.1 × 10^4	[9.7 × 10^3, 3.5 × 10^4]
HMML /MAE	k_{het}, s^{-1}	6.1 × 10^{-7}	[5.2 × 10^{-7}, 7.1 × 10^{-7}]	2.8 × 10^{-7}	[1.8 × 10^{-7}, 4.0 × 10^{-7}]
	β_{OS}	0.11	[0.09, 0.12]	0.05	[0.03, 0.07]
	$k_{H_2O,H^+}H_{aq}$, M^{-1} atm^{-1} s^{-1}	1.5 × 10^3	[8.1 × 10^2, 2.4 × 10^3]	6.8 × 10^2	[5.7 × 10^2, 7.8 × 10^2]
	$k_{H_2O,HSO_4^-}H_{aq}$, M^{-1} atm^{-1} s^{-1}	3.2 × 10^3	[1.7 × 10^3, 4.9 × 10^3]	1.3 × 10^3	[7.6 × 10^2, 2.1 × 10^3]
	$k_{SO_4^{2-},H^+}H_{aq}$, M^{-1} atm^{-1} s^{-1}	8.0 × 10^2	[5.4 × 10^2, 1.1 × 10^3]	4.9 × 10^2	[3.1 × 10^2, 6.8 × 10^2]

Number of bootstrap replications = 1000

Text S3. Data selected for estimations

This study selects only data from summer daytime hours from 12:00 to 16:00 (local time) for estimating the k_{het} and γ values due to four reasons. First, the abundances of isoprene and its oxidation products during cold season were significantly lower than those observed during warm season (Table S3, Figure S10). Winter data could lead to larger uncertainties in constraining uptake coefficients. Second, the OBM results show that isoprene oxidation products were more influenced by regional transport in cold season (Figure S11). In comparison, transport effect played a relatively minor role in regulating their abundances in warm season and was almost negligible during summer daytime (Figure S12). Third, OH oxidation is the predominant channel for isoprene SOA formation during daytime, while other pathways (e.g., oxidation by NO_3 radical or O_3) can be much more competitive at night.[10-13] The distinct diurnal patterns observed in isoprene, MACR+MVK (Figure S13), MTLs and MGA (Figure S14), with peak values occurring around noontime, along with the contrasting patterns seen in the modeled isoprene epoxides (Figure S15), provide additional evidence supporting the notion that local photooxidation processes via epoxide pathways are the primary drivers of MTLs and MGA formation during summer daytime hours. Lastly, based on the ECMWF reanalysis data and the sensitivity analysis given in Text S5, we found that by selecting a subset data of the afternoon hours from 12:00 to 16:00 (local time), the influence of background air entrainment due to PBL height growth on k_{het} is small. Therefore, focusing on summer daytime data from 12:00 to 16:00 (local time) for k_{het} and γ estimations can help minimize the impacts from regional transport and other chemical formation pathways. This approach, in turn, serves to reduce the uncertainties associated with these estimations.

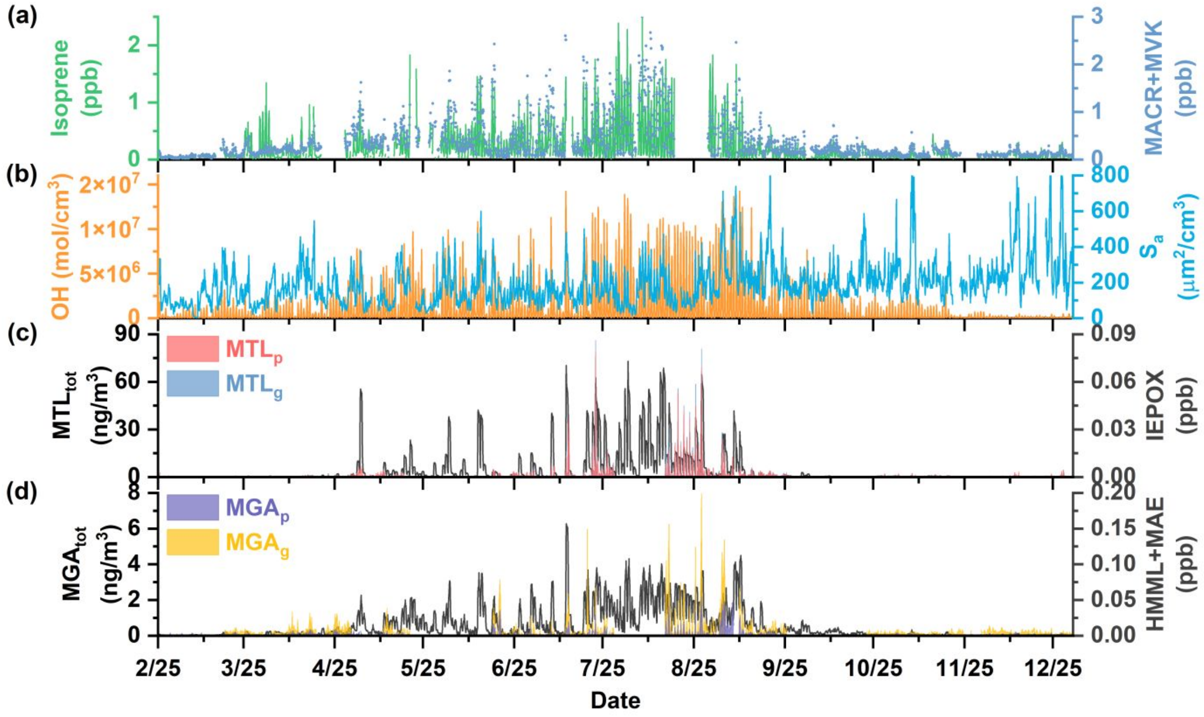

Figure S10. Bi-hourly variations of (a) isoprene and its early-generation oxidation products

(MACR+MVK) measured by GC-FID; (b) OBM-modeled OH radicals and particle surface area (S_a) obtained from SMPS measurements; (c) TAG-measured particle-phase MTLs (MTL_p) and its gas-phase (MTL_g) concentrations estimated by gas-particle partitioning equilibrium theory, and OBM-simulated gas-phase precursor IEPOX (dark line); and (d) TAG-measured particle-phase MGA (MGA_p) and its gas-phase (MGA_g) concentrations estimated by gas-particle partitioning equilibrium theory, and OBM-simulated gas-phase precursor HMML+MAE (dark line).

Table S3. Monthly average concentrations (Avg ± SD) of isoprene and its oxidation products observed at SAES site or estimated by observation-based model (OBM) and particle-gas phase partitioning equilibrium theory.

Month (2020)	Isoprene[a] (ppb)	MACR+MVK[a] (ppb)	IEPOX[b] (ppt)	HMML+MAE[b] (ppt)	MTL_p[c] (ng/m^3)	MGA_p[c] (ng/m^3)	MTL_g[d] (ng/m^3)	MGA_g[d] (ng/m^3)
March	0.09 ± 0.14	0.12 ± 0.09	0.04 ± 0.06	1.23 ± 1.22	0.11 ± 0.14	0.04 ± 0.03	0.03 ± 0.03	0.14 ± 0.06
April	0.15 ± 0.20	0.24 ± 0.10	0.24 ± 0.36	4.08 ± 2.64	0.36 ± 0.27	0.07 ± 0.05	0.13 ± 0.13	0.26 ± 0.19
May	0.26 ± 0.25	0.51 ± 0.25	4.06 ± 8.32	16.9 ± 13.0	0.85 ± 1.15	0.08 ± 0.05	0.11 ± 0.09	0.23 ± 0.20
June	0.29 ± 0.28	0.51 ± 0.44	5.07 ± 9.59	17.3 ± 19.6	1.17 ± 1.06	0.14 ± 0.25	0.07 ± 0.10	0.23 ± 0.34
July	0.36 ± 0.41	0.62 ± 0.53	10.8 ± 15.1	30.5 ± 30.2	2.49 ± 2.04	0.15 ± 0.13	0.25 ± 0.24	0.28 ± 0.22
August	0.54 ± 0.53	0.99 ± 0.67	18.6 ± 18.1	46.1 ± 24.8	5.24 ± 4.25	0.21 ± 0.35	1.17 ± 1.09	0.69 ± 1.06
September	0.20 ± 0.25	0.38 ± 0.33	3.79 ± 7.52	22.2 ± 24.5	1.87 ± 2.43	0.26 ± 0.38	0.23 ± 0.29	0.39 ± 0.57
October	0.08 ± 0.06	0.18 ± 0.10	0.21 ± 0.39	3.92 ± 3.37	0.43 ± 0.27	0.04 ± 0.03	0.16 ± 0.09	0.15 ± 0.11
November	0.07 ± 0.06	0.14 ± 0.09	0.05 ± 0.08	1.36 ± 1.19	0.37 ± 0.30	0.03 ± 0.03	0.09 ± 0.09	0.11 ± 0.07
December	0.06 ± 0.05	0.11 ± 0.05	0.03 ± 0.03	0.95 ± 0.68	0.33 ± 0.39	0.03 ± 0.03	0.07 ± 0.08	0.11 ± 0.09

[a] Isoprene and MACR+MVK concentrations were calculated based on GC-FID online measurement. [b] IEPOX and HMML+MAE concentrations were derived from the OBM model. [c] MTL_p and MGA_p concentrations were particle-phase concentrations measured by the TAG system. [d] MTL_g and MGA_g were gas-phase concentrations estimated based on absorptive gas-organic matter partitioning theory and gas-aqueous phase partitioning equilibrium using the TAG online measurement of particle phase concentrations of the two tracers (see Text S4).

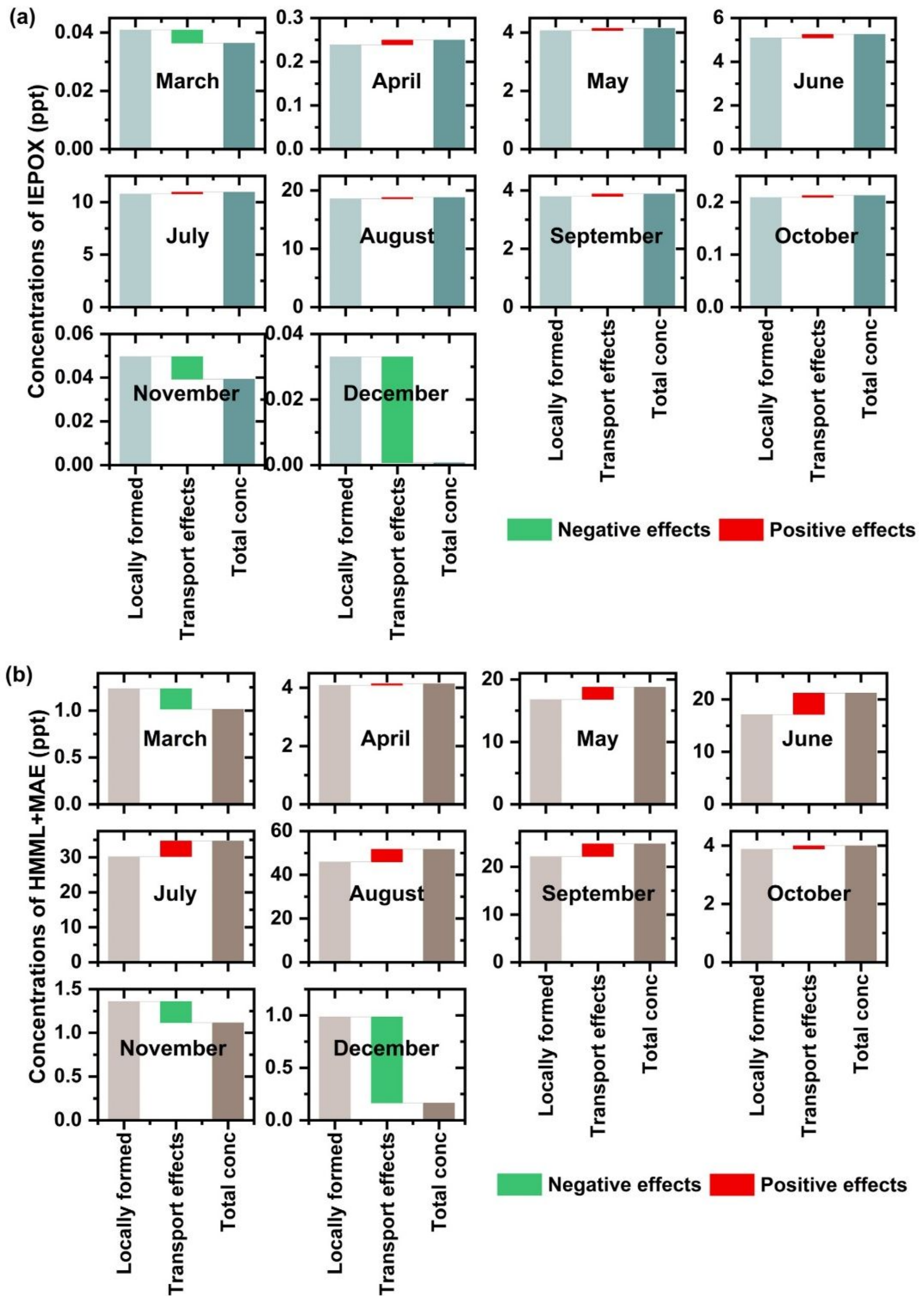

Figure S11. Estimated regional transport contributions to monthly averaged concentrations of (a) IEPOX and (b) HMML+MAE.

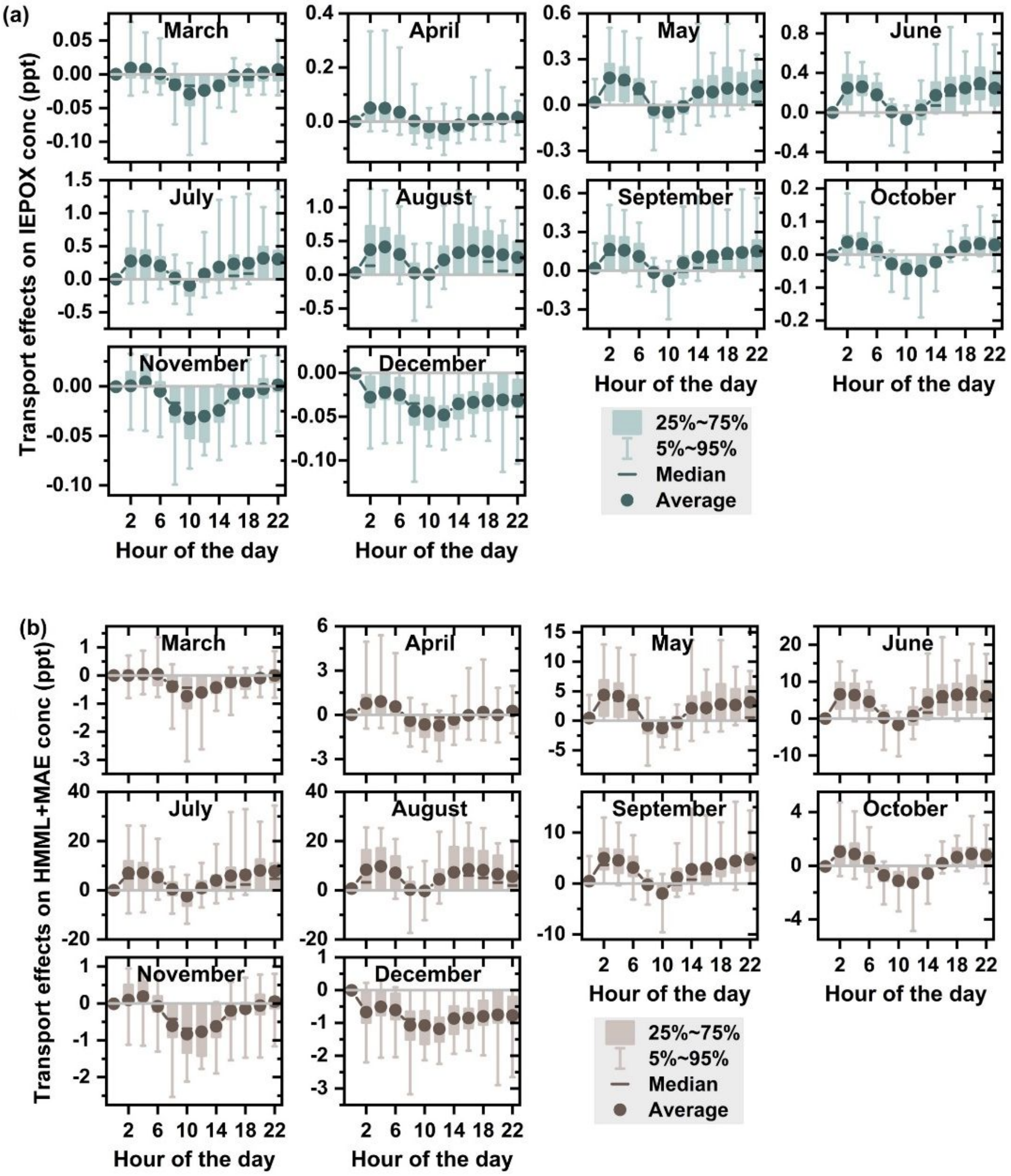

Figure S12. Estimated regional transport contributions to monthly average diurnal variations of (a) IEPOX and (b) HMML+MAE concentrations.

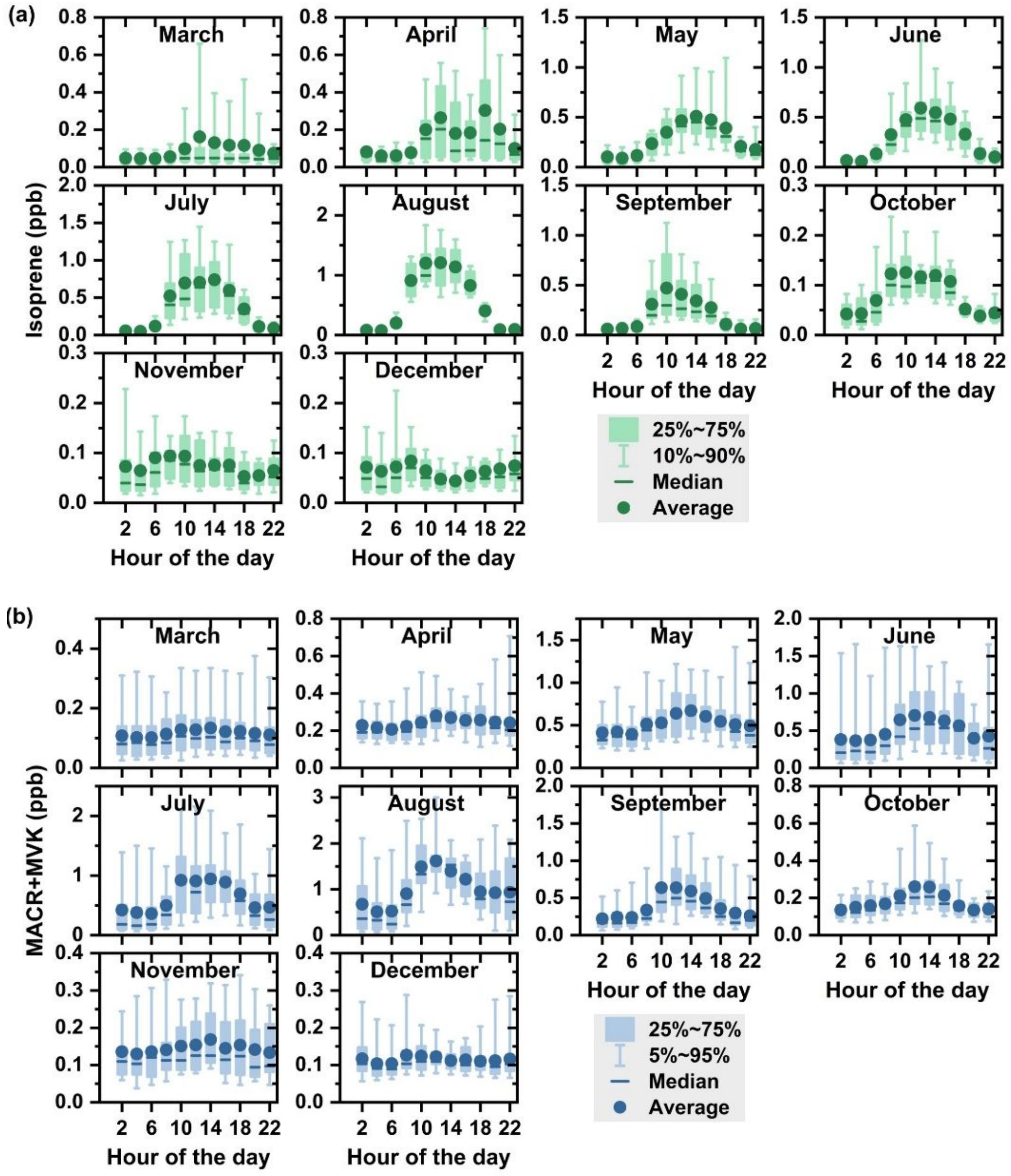

Figure S13. Observed monthly average diurnal variations of (a) isoprene and (b) its first-generation oxidation products (MACR+MVK) concentrations.

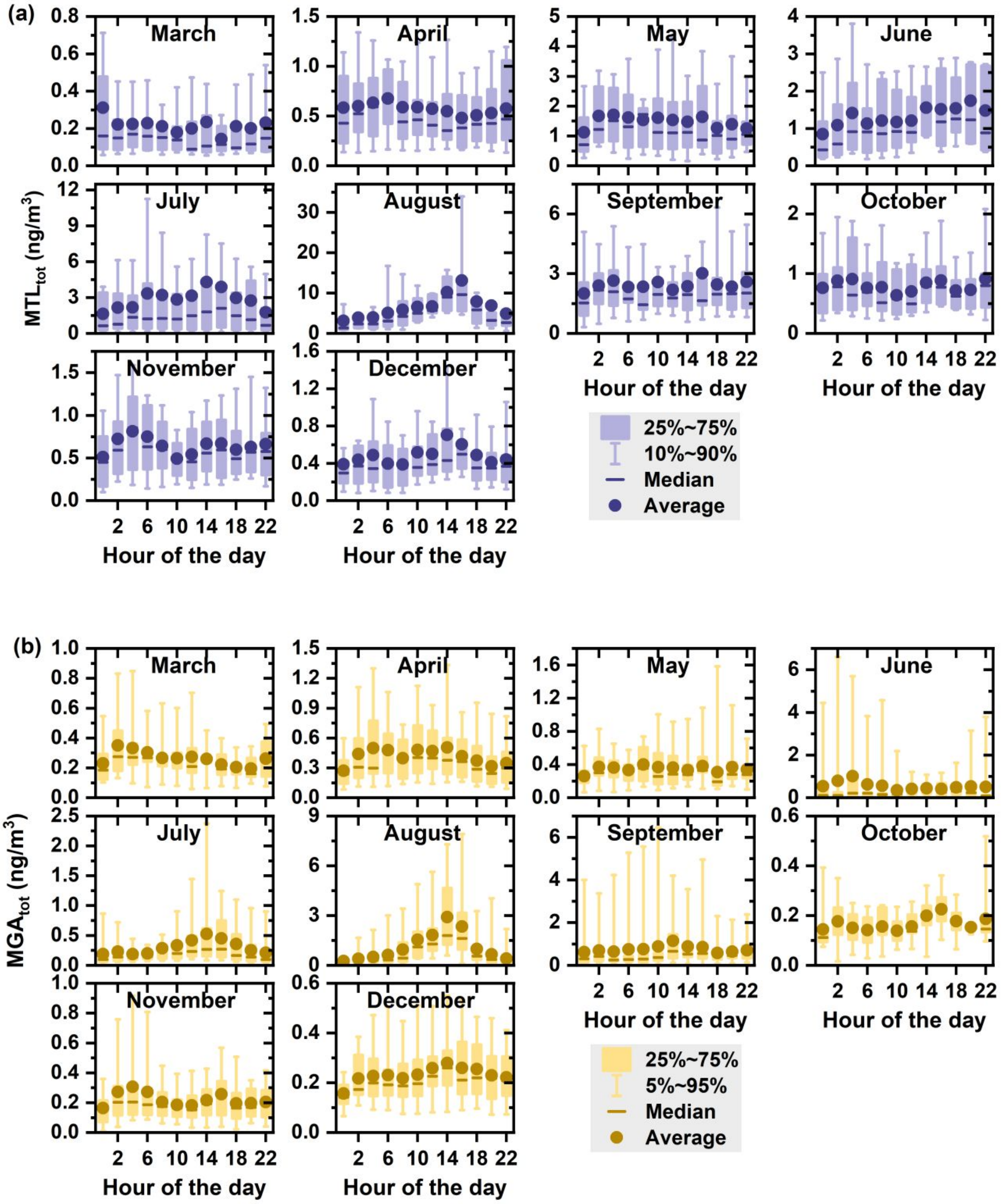

Figure S14. Observed monthly average diurnal variations of (a) MTLs and (b) MGA total concentrations.

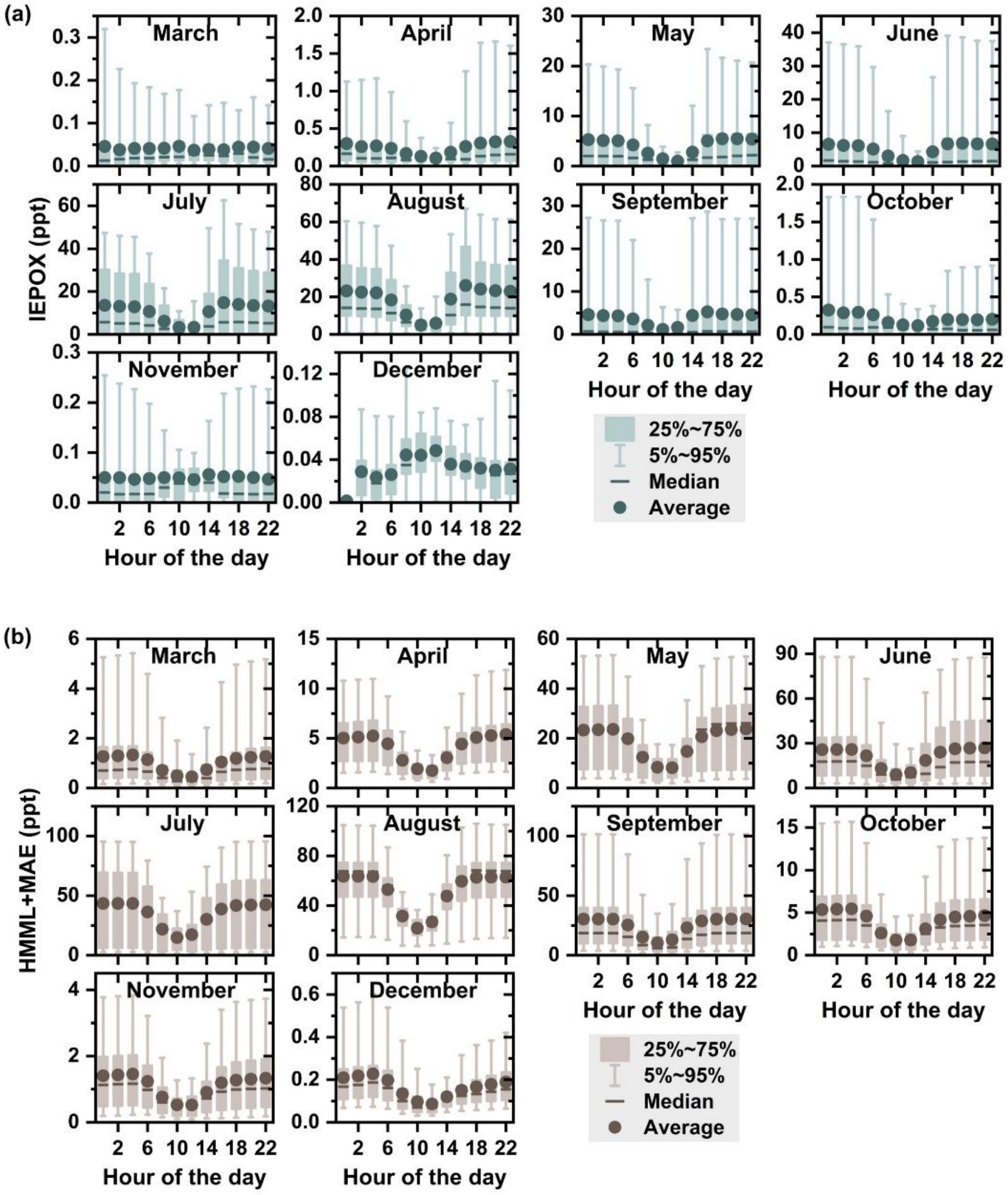

Figure S15. Monthly average diurnal variations of (a) IEPOX and (b) HMML+MAE concentrations from the observation-based model.

Text S4. Estimating aqueous-, organic-, and gas- phase concentrations of MTLs and MGA

In this study, the TAG system only measured the particle-phase concentrations ($C_{particle}$), which can be regarded as the sum of concentrations in aqueous-phase (C_{aq}) and organic-phase (C_{org}). Since MTLs and MGA are semi-volatile, it is necessary to consider their gas-aqueous-organic partitioning. The C_{aq}, C_{org}, and gas-phase concentrations (C_{gas}) when reaching partitioning equilibrium can be determined from the equations (S5) - (S7), which are based on equilibrium partitioning and mass conservation:

$$C_{gas} = \frac{C_{org}}{K_{OM}C_{OM}} \quad \text{(S5)}$$

$$\frac{C_{aq}}{C_{gas}} = f_H \quad \text{(S6)}$$

$$C_{aq} + C_{org} = C_{particle} \quad \text{(S7)}$$

The following three equations, (S8)-(S10), can be derived from equations (S5)-(S7),

$$C_{gas} = \frac{1}{K_{OM}C_{OM} + f_H}C_{particle} \quad \text{(S8)}$$

$$C_{org} = \frac{K_{OM}C_{OM}}{K_{OM}C_{OM} + f_H}C_{particle} \quad \text{(S9)}$$

$$C_{aq} = \frac{f_H}{K_{OM}C_{OM} + f_H}C_{particle} \quad \text{(S10)}$$

where C_{OM} (μg/m^3) is the mass concentration of organic matters (OM) in the particle phase, which is estimated by multiplying the measured organic carbon (OC) with a conversion factor of 1.8.[14] K_{OM} (m^3/μg) is an absorptive partitioning coefficient defined by equation (S11),

$$K_{OM} = \frac{RT}{10^6 p_L^0 \xi_{OM} \overline{MW_{OM}}} \quad \text{(S11)}$$

where R (m^3 Pa K^{-1} mol^{-1}) is the ideal gas constant, T (K) is the ambient temperature, $\overline{MW_{OM}}$ is the mean molecular weight of the absorbing OM assumed to be 200 g mol^{-1},[15] ξ_{OM} is the activity coefficient of each compound in the absorbing OM (assumed to be unity), and p_L^0 is the sub-cooled vapor pressure (Pa). In our study, a value of 1.81×10^{-4} Pa and 3.76×10^{-4} Pa is adopted for MTLs and MGA, respectively.[16]

The unitless gas-aqueous phase partitioning coefficient (f_H) of MTLs and MGA derived from the Henry's Law can be estimated using equation (S12),

$$f_H = 10^{-6} H_{aq} RT[H_2O] \quad \text{(S12)}$$

where H_{aq} (M atm^{-1}) is the effective Henry's law constant. In this study, a value of 3.38 × 10^{10} M atm^{-1} and 5.25 × 10^8 M atm^{-1} is adopted for MTLs and MGA, respectively.[16] [H_2O] is the aerosol liquid water content (ALWC) (M) obtained from ISORROPIA-II[17,18].

Text S5. Sensitivity analysis of impacts of planetary boundary layer on k_{het} estimations

The growth of planetary boundary layer (PBL) height during daytime can affect ground-level pollutant concentrations. To examine the impacts of PBL growth on k_{het} estimations and determine the data set that are less influenced by boundary layer height. Equations (1) and (2) in the main text are revised as equations (S13) and (S14), respectively:

$$\frac{\Delta[MTLs_{tot}]}{\Delta t} = (1-\beta_{MTL-OS})k_{het_{IEPOX}}[IEPOX] - k_{OH+MTLs}[OH_{gas}]\,[MTLs_{gas}] + \frac{1}{H}\frac{\Delta H}{\Delta t}([MTLs_{tot}]_{bkg} - [MTLs_{tot}]),\quad if\ \frac{\Delta H}{\Delta t} > 0 \qquad \text{(S13)}$$

$$\frac{\Delta[MGA_{tot}]}{\Delta t} = (1-\beta_{MGA-OS})k_{het_{HMML/MAE}}[HMML + MAE] - k_{OH+MGA}[OH_{gas}]\,[MGA_{gas}] + \frac{1}{H}\frac{\Delta H}{\Delta t}([MGA_{tot}]_{bkg} - [MGA_{tot}]),\quad if\ \frac{\Delta H}{\Delta t} > 0 \qquad \text{(S14)}$$

where $\frac{1}{H}\frac{\Delta H}{\Delta t}([MTLs_{tot}]_{bkg} - [MTLs_{tot}])$ and $\frac{1}{H}\frac{\Delta H}{\Delta t}([MGA_{tot}]_{bkg} - [MGA_{tot}])$ are used to account for the changes in ground-level concentrations due to the growth of the planetary boundary layer (PBL) height (H) during the day. $[MTLs_{tot}]_{bkg}$ and $[MTLs_{tot}]_{bkg}$ are their concentrations in the background air above the PBL. Since $[MTLs_{tot}]_{bkg}$ and $[MGA_{tot}]_{bkg}$ are unknown, we select data from daytime hours when PBL height were decreasing or slowly increasing for subsequent analysis. The hourly PBL height at the observation site was downloaded from ECMWF reanalysis version 5 (ERA5) (https://cds.climate.copernicus.eu/cdsapp#!/dataset/reanalysis-era5-single-levels?tab=overview). As illustrated in Figure S16, the PBL height increased rapidly during the morning hours, then the rate of growth significantly slowed around noontime (12:00 p.m.) and turned negative afterwards. Consequently, it is expected that the afternoon observation data would be less susceptible to the impact of neglecting the PBL growth terms.

To investigate the potential biases in the k_{het} estimations from neglecting the PBL growth term and selecting data from different daytime periods, two sets of k_{het} estimates were performed, one with and one without the PBL growth term. In the second set of the estimation, $[MTLs_{tot}]_{bkg}$ and $[MGA_{tot}]_{bkg}$ were set to zero to represent the maximum impact of the PBL growth term. Figure S17 shows that including all daytime data (6:00-16:00 local time) would lead to large differences between the two sets of estimation. The agreement between the two sets of results is the best using the afternoon data (12:00-16:00). Using this subset of data and ignoring the PBL growth term would only lead to small over-predictions of k_{het} for HMML/MAE (< 15%) and IEPOX (<8%). Thus, the afternoon data was used for the detailed analysis in this study.

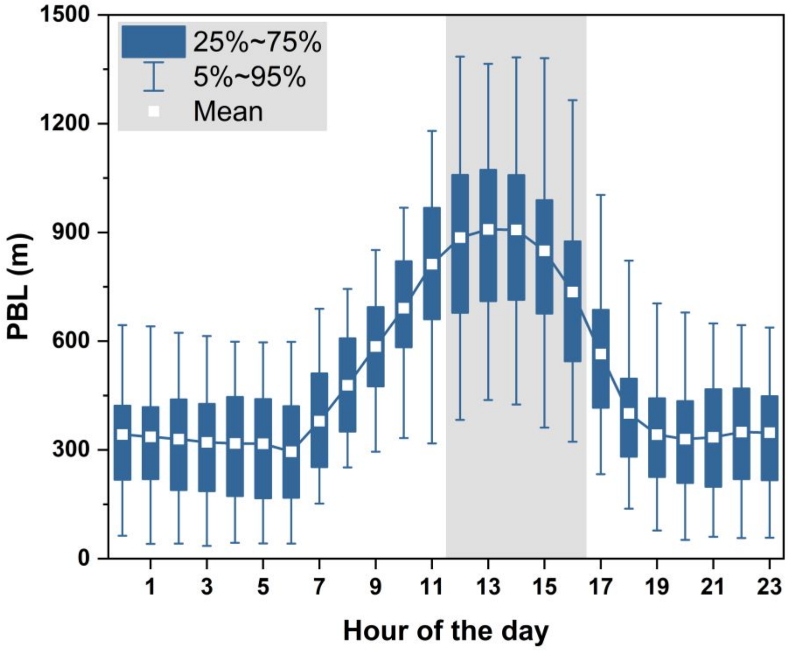

Figure S16. Diurnal variations of PBL height at SAES site. Hours (12:00 ~ 16:00) selected for estimations are shadowed by grey color.

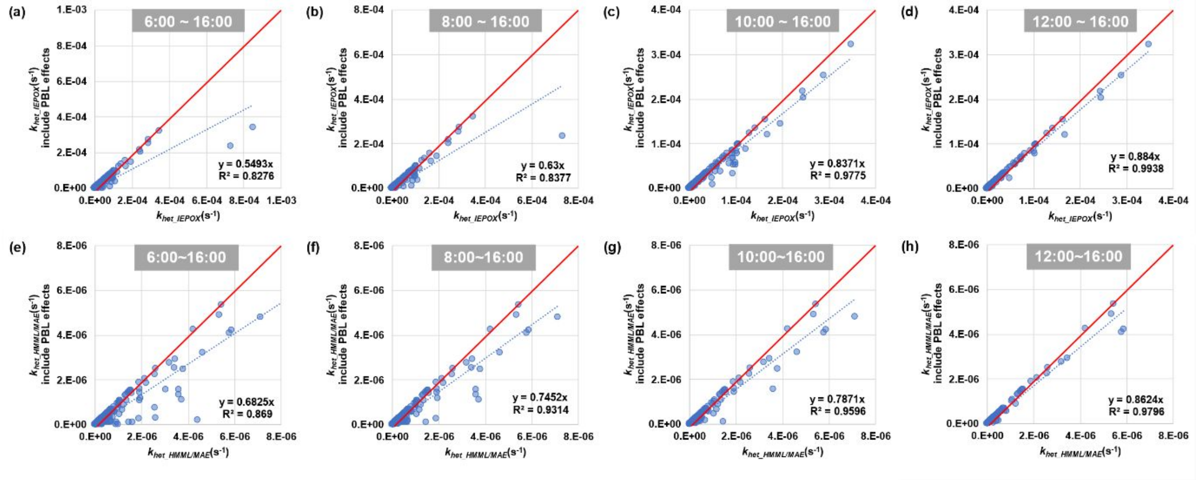

Figure S17. Effects of PBL growth on estimated heterogeneous reaction rates of (a-d) IEPOX (k_{het_IEPOX}) and (e-h) HMML/MAE ($k_{het\_HMML/MAE}$) with different daytime hours selected. Red lines are 1:1 lines.

Text S6. Estimation of the organosulfate formation branching ratios

Reactive uptake of IEPOX (or MAE/HMML) produces both MTLs (or MGA) and organosulfates in the particle phase. The ratio of organosulfate formation rate to the loss rate of IEPOX (or MAE) due to reactive uptake is defined as the organosulfate (β_{OS}) branching ratio. However, β_{OS} cannot be directly determined during the campaign due to the lack of online measurements of organosulfates. In this study, the β_{OS} values in Equations (1) and (2) are determined using an iterative approach. In this approach, an initial estimation of β_{OS} (e.g., 0.5) is applied in Equations (1) or (2) to determine k_{het}. After that, the uptake coefficient γ, the pseudo-first order aqueous reaction rate coefficient k_{aq} (as $k_{aq}H_{aq}$), and the third-order reaction rate coefficients k_{H_2O,H^+}, k_{H_2O,HSO_4^-}, and $k_{SO_4^{2-},H^+}$ ($k_{H_2O,H^+}H_{aq}$, $k_{H_2O,HSO_4^-}H_{aq}$, and $k_{SO_4^{2-},H^+}H_{aq}$) are determined using Equations (3) - (6), respectively. The aqueous reaction parameters are used to provide an updated estimation of β_{OS}, as shown in Equation (S15), which assumes that reactions with sulfate as the nucleophile produces organosulfates while the reactions with water as the nucleophile generate MTLs (or MGA).

$$\beta_{OS} = \frac{k_{SO_4^{2-},H^+}H_{aq}[SO_4^{2-}][H^+]}{k_{SO_4^{2-},H^+}H_{aq}[SO_4^{2-}]\,[H^+] + k_{H_2O,HSO_4^-}H_{aq}[H_2O]\,[HSO_4^-] + k_{H_2O,H^+}H_{aq}[H_2O]\,[H^+]} \quad \text{(S15)}$$

With the updated β_{OS}, the aqueous reaction parameters can be recalculated using the approach described above. The iteration process is summarized in Figure S18. The iteration stops when the relative changes in β_{OS} and the aqueous parameters are less than 0.05.

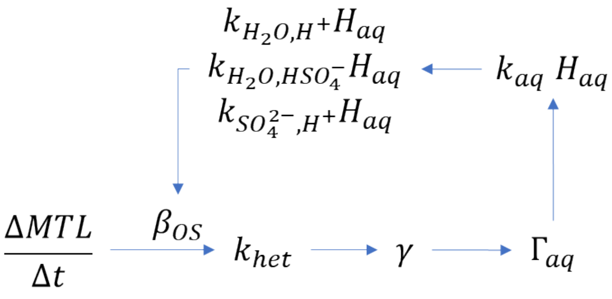

Figure S18. Illustration of the iterative approach to determine the organosulfate branching ratio (β_{OS}).

The iterative calculation results are given in Table S4. For IEPOX, the changes of all its estimated kinetic data were smaller than 0.05 after the 7th iteration. Therefore, the kinetic data obtained in this iterative solution were adopted as final estimation results for IEPOX. For HMML/MAE, its estimated $\beta_{MGA\text{-}OS}$ and three reaction rate constants showed negligible changes after the 6th iteration. Thus, the kinetic data obtained in this iterative solution were adopted as final estimation results for HMML/MAE.

335

Table S4. Aqueous-phase reaction coefficients of isoprene epoxides and the average organosulfate formation pathway branching ratios (β_{OS}) derived from multivariate linear regression (MLR) analysis in each iteration step. The results adopted for further analysis are highlighted in bold.

Isoprene epoxides	iteration	Reaction constants (M^{-1} atm^{-1} s^{-1})			β_{OS}	R^a	Changes in k^{th} iteration compared with $(k-1)^{th}$ iteration				
		$k_{H_2O,H^+}H_{aq}$	$k_{H_2O,HSO_4^-}H_{aq}$	$k_{SO_4^{2-},H^+}H_{aq}$			$\Delta k_{H_2O,H^+}H_{aq}$	$\Delta k_{H_2O,HSO_4^-}H_{aq}$	$\Delta k_{SO_4^{2-},H^+}H_{aq}$	$\Delta\beta_{OS}$	ΔR
IEPOX	1st	1.25E+05	4.83E+05	1.32E+05	0.069	0.613					
	2nd	9.00E+04	3.34E+05	1.02E+05	0.089	0.700	-0.28	-0.31	-0.23	0.28	0.14
	3rd	7.22E+04	2.72E+05	8.33E+04	0.097	0.742	-0.19	-0.19	-0.18	0.09	0.06
	4th	6.45E+04	2.44E+05	7.40E+04	0.102	0.756	-0.11	-0.10	-0.11	0.05	0.02
	5th	5.90E+04	2.21E+05	6.75E+04	0.104	0.765	-0.08	-0.09	-0.08	0.02	0.01
	6th	5.75E+04	2.07E+05	6.53E+04	0.105	0.768	-0.03	-0.06	0.03	0.01	0.00
	**7th**	**5.85E+04**	**2.11E+05**	**6.65E+04**	**0.106**	**0.769**	**0.02**	**0.02**	**0.02**	**0.01**	**0.00**
	8th	5.87E+04	2.10E+05	6.54E+04	0.105	0.769	0.00	0.00	-0.01	-0.01	0.00
HMML/ MAE	1st	2.90E+03	6.25E+03	1.46E+03	0.062	0.636					
	2nd	2.33E+03	5.14E+03	1.11E+03	0.079	0.729	-0.20	-0.18	-0.24	0.27	0.15
	3rd	1.98E+03	4.43E+03	9.47E+02	0.088	0.780	-0.15	-0.14	-0.15	0.11	0.07
	4th	1.73E+03	3.85E+03	8.37E+02	0.095	0.790	-0.12	-0.13	-0.12	0.07	0.02
	5th	1.61E+03	3.50E+03	7.57E+02	0.099	0.794	-0.07	-0.09	-0.10	0.04	0.00
	**6th**	**1.56E+03**	**3.41E+03**	**7.33E+02**	**0.100**	**0.795**	**-0.03**	**-0.03**	**-0.03**	**0.01**	**0.00**
	7th	1.55E+03	3.40E+03	7.34E+02	0.099	0.795	-0.01	0.00	0.00	-0.01	0.00

[a] Correlation coefficient of multilinear regression.

Table S5. Comparison of mean organosulfate formation molar branching ratios estimated in this study and reported in previous field campaigns.

Reference	Location	$\beta_{MTL\text{-}OS}$	$\beta_{MGA\text{-}OS}$	Concentration of SO_4^{2-} and particle pH
This study	Shanghai, China	0.11 ± 0.10	0.10 ± 0.10	$[SO_4^{2-}]$ = 4.6 μg/m^3, pH ~ 3.5
Zhang et al., 2022 [19]	Hefei, China	0.02	0.19	$[SO_4^{2-}]$ = 11.1 μg/m^3, pH ~ 4.2
Zhang et al., 2022 [19]	Beijing, China	0.04	0.18	$[SO_4^{2-}]$ = 8.3 μg/m^3, pH ~ 4.3
Zhang et al., 2022 [19]	Kunming, China	0.03	0.22	$[SO_4^{2-}]$ = 7.1 μg/m^3, pH ~ 4.0
He et al., 2018 [20]	Guangzhou, China	0.01	0.10	$[SO_4^{2-}]$ = 23.0 μg/m^3, pH ~ 0.5
Budisulistiorini et al., 2015 [21]	Tennessee, U.S.	0.40	0.44	$[SO_4^{2-}]$ = 1.9 μg/m^3
Rattanavaraha et al., 2016 [22]	Alabama, U.S.	0.27	0.29	$[SO_4^{2-}]$ = 2.0 μg/m^3, pH ~ 1.8
Lin et al., 2012 [23]	Georgia, U.S.	0.12	/	/
Cui et al., 2018 [24]	Manaus, Brazil	0.64	/	/

Text S7. Sensitivity analysis of impacts of aqueous-phase loss pathway on k_{het} estimations

In addition to gas-phase oxidation by OH radicals, aqueous-phase reactions with OH also represent a potential removal pathway for MTLs and MGA. However, the modeled aqueous-phase OH has large uncertainties. Thus, we include a sensitivity analysis to examine whether omitting the aqueous-phase removal process will lead to considerable uncertainties in estimating the pseudo first-order heterogeneous reaction rate coefficients (k_{het}) for IEPOX and HMML/MAE. Equations (1) and (2) in the main text are revised to include the loss of the organic marker compounds due to reactions with aqueous OH radicals:

$$\frac{\Delta[MTLs_{tot}]}{\Delta t} = (1-\beta_{MTL-OS})k_{het\_IEPOX}[IEPOX] - k_{OH+MTLs}[OH]\,[MTLs_{gas}] - k_{OH+MTLs\_aq}[OH_{aq}]\,[MTLs_{aq}] \quad \text{(S16)}$$

$$\frac{\Delta[MGA_{tot}]}{\Delta t} = (1-\beta_{MGA-OS})k_{het_{HMML/MAE}}[HMML+MAE] - k_{OH+MGA}[OH][MGA_{gas}] - k_{OH+MGA\_aq}[OH_{aq}]\,[MGA_{aq}] \quad \text{(S17)}$$

where $k_{OH+MTLs\_aq}$ and k_{OH+MGA_aq} are the aqueous-phase reaction rate coefficients of MTLs with OH radicals. The values of the two aqueous rate coefficients are taken from Zhang et al. (2023)[16]. $[MTL_{aq}]$ and $[MGA_{aq}]$ are aqueous-phase molar concentrations of MTLs and MGA, respectively. $[OH_{aq}]$, the OH radical concentration in aqueous phase, is estimated by multiplying the equilibrium concentration of OH radical in the aqueous phase based on the gas phase OH and its Henry's Law constant with a scaling factor of 0.005, which accounts for the loss of OH due to dissolved species.[9]

Figure S19 shows that estimated k_{het} values with and without the consideration of aqueous-phase loss pathways have strong correlations. For HMML/MAE, the slope of the linear regression is close to 1, indicating that aqueous-phase loss pathway is likely unimportant. Since MTLs have a larger mass fraction (~76%) partitioned in the aqueous phase, including aqueous-phase removal process leads to a 25 ± 30% increase in estimated k_{het_IEPOX}. This analysis suggests that the values of the heterogeneous reaction coefficients are not significantly influenced by the aqueous loss. Therefore, aqueous-phase removals by OH were not considered in the later analysis and discussion.

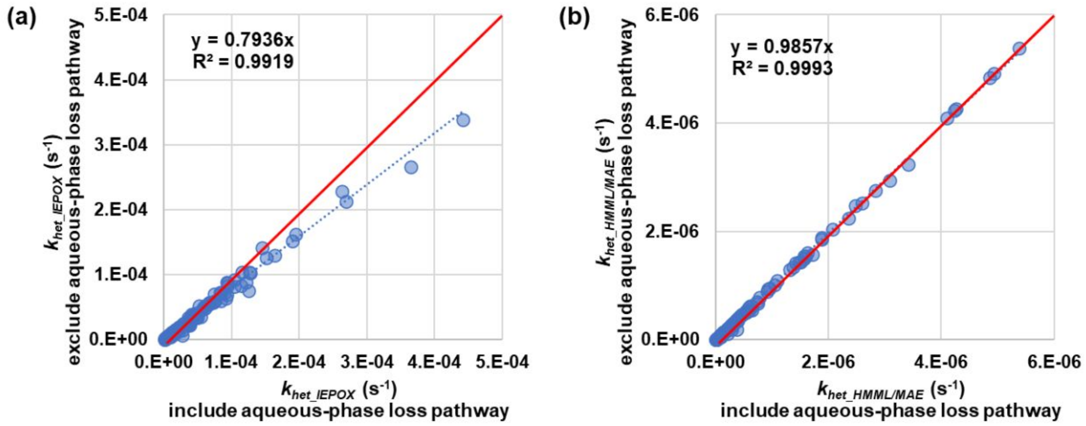

Figure S19. Correlations between estimated heterogeneous reaction rate coefficients of (a) IEPOX (k_{het_IEPOX}) and (b) HMML/MAE ($k_{het\_HMML/MAE}$) with and without considering the aqueous-phase loss due to reactions with the OH radicals. Blue dashed lines are determined using linear least-squares regression by forcing a zero intercept. Red lines are 1:1 lines.

Text S8. Sensitivity analysis of impacts of deposition loss pathway on k_{het} estimations

The abundances of MTLs and MGA in the atmosphere are also affected by dry/wet deposition. However, their deposition rates under ambient conditions are not available. Here we conduct a sensitivity analysis to examine whether excluding the deposition loss pathway will lead to large uncertainties in our k_{het} estimations for IEPOX and HMML/MAE. Equations (1) and (2) in the main text are revised to include the deposition loss pathway:

$$\frac{\Delta[MTLs_{tot}]}{\Delta t} = (1-\beta_{MTL-OS})k_{het\_IEPOX}[IEPOX] - k_{OH+MTLs}[OH]\,[MTLs_{gas}] - \frac{v_d}{H}[MTLs_p] - \frac{v'_d}{H}[MTLs_{gas}] \quad \text{(S18)}$$

$$\frac{\Delta[MGA_{tot}]}{\Delta t} = (1-\beta_{MGA-OS})k_{het_{HMML/MAE}}[HMML+MAE] - k_{OH+MGA}[OH][MGA_{gas}] - \frac{v_d}{H}[MGA_p] - \frac{v'_d}{H}[MGA_{gas}] \quad \text{(S19)}$$

where v_d and v'_d are dry deposition velocities in the particle phase and gas phase, respectively. Although v_d for MTLs and MGA are unknown, the deposition velocities for $PM_{2.5}$ and its major components (e.g., sulfate, nitrate, calcium) have been measured and reported in the literature with values ranging from 0.01-10 cm s^{-1}.[25-27] Here we assumed a deposition rate equivalent to 10 cm s^{-1} for v_d, almost an upper bound for $PM_{2.5}$ species. A value of 2.5 cm s^{-1} is adopted for v'_d, which is assumed to be the deposition rate of IEPOX in the atmosphere in a few studies[28,29]. Figure S20 shows that estimated k_{het} values with and without the consideration of deposition loss have strong linear correlations with slopes around 0.67. Since the v_d and v'_d values adopted here almost represent an upper bound for MTLs and MGA in the atmosphere, thus excluding deposition removal process would result in a decrease of less than 33% in estimated k_{het}.

Figure S21 presents the scatter plots of daytime concentrations of MTLs and MGA with consumed amounts of isoprene, which was estimated based on photochemical age with equations (S20) - (S21)[30,31]. The daytime concentrations of MTLs and MGA in particle phase were well correlated with consumed amounts of isoprene with $R > 0.50$, reaffirming that the daytime concentrations of MTLs and MGA were mainly affected by acid-driven heterogeneous process, and other formation or loss pathways (e.g., deposition loss) might play a relatively minor role in regulating their mass abundances. Therefore, deposition removals were not considered in the later analysis and discussions.

$$[Isoprene]_{consumed} = Isoprene_t \times \left(\exp\left(k_{isoprene+OH}[OH]\Delta t\right) - 1\right) \quad (20)$$

$$[OH]\Delta t = \frac{1}{k_x - k_B} \times \left(ln\frac{[X]}{[B]}\bigg|_{t=t_0} - ln\frac{[X]}{[B]}\bigg|_{t=t} \right) \quad (21)$$

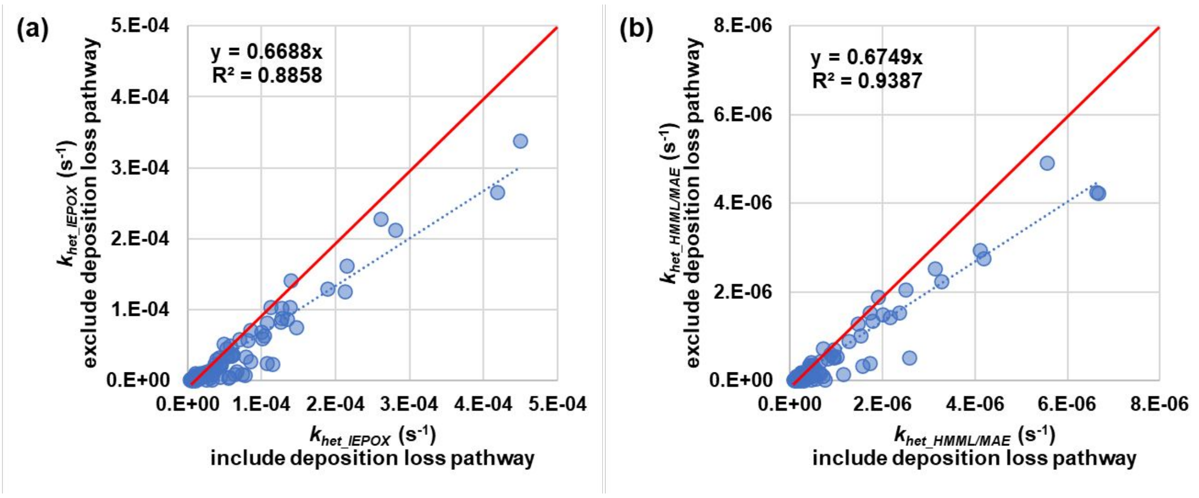

Figure S20. Correlations between estimated heterogeneous reaction rate coefficients of (a) IEPOX (k_{het_IEPOX}) and (b) HMML/MAE ($k_{het\_HMML/MAE}$) with and without considering the deposition losses. Blue dashed lines are determined using linear least-squares regression by forcing a zero intercept. Red lines are 1:1 lines.

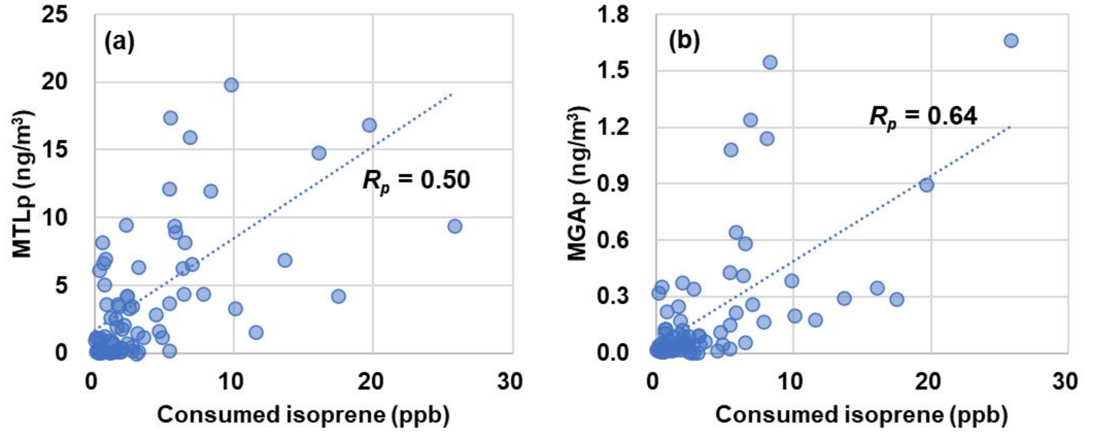

Figure S21. Correlations between consumed isoprene and particle-phase concentrations of (a) MTLs and (b) MGA.

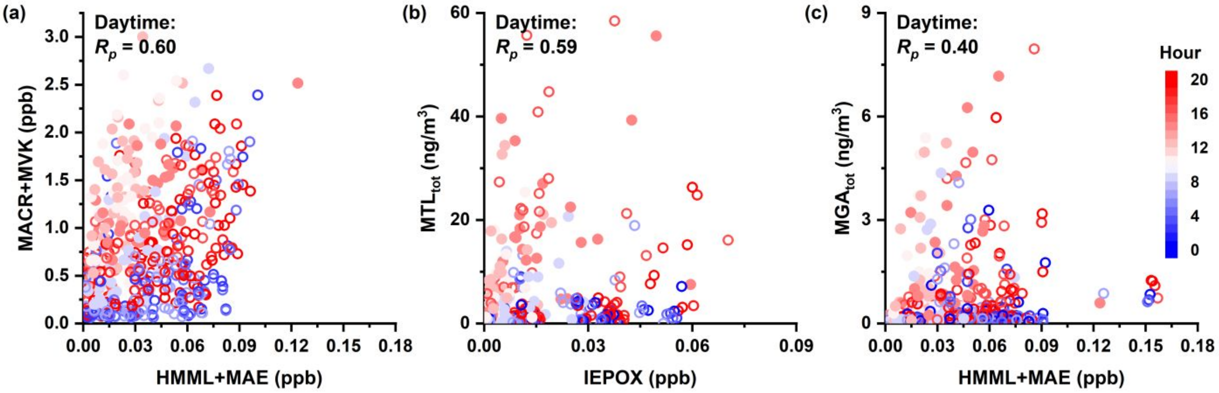

Figure S22. Correlations of (a) HMML+MAE and MACR+MVK, (b) IEPOX and MTLs, (c) HMML+MAE and MGA in summer. Dots are colored by hour of the day and solid dots represent daytime data set.

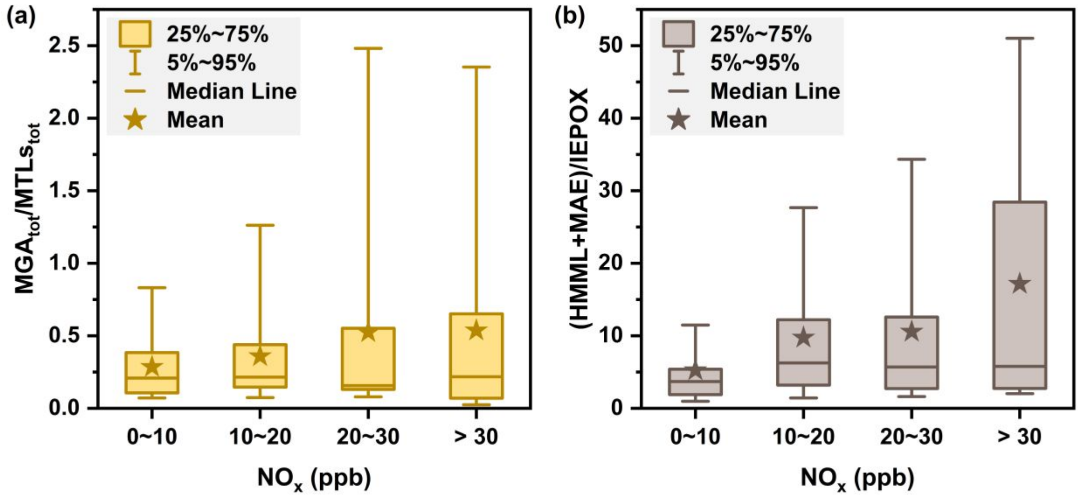

Figure S23. Ratios of (a) MGA/MTLs and (b) (HMML+MAE)/IEPOX as a function of NO_x based on summer daytime data set.

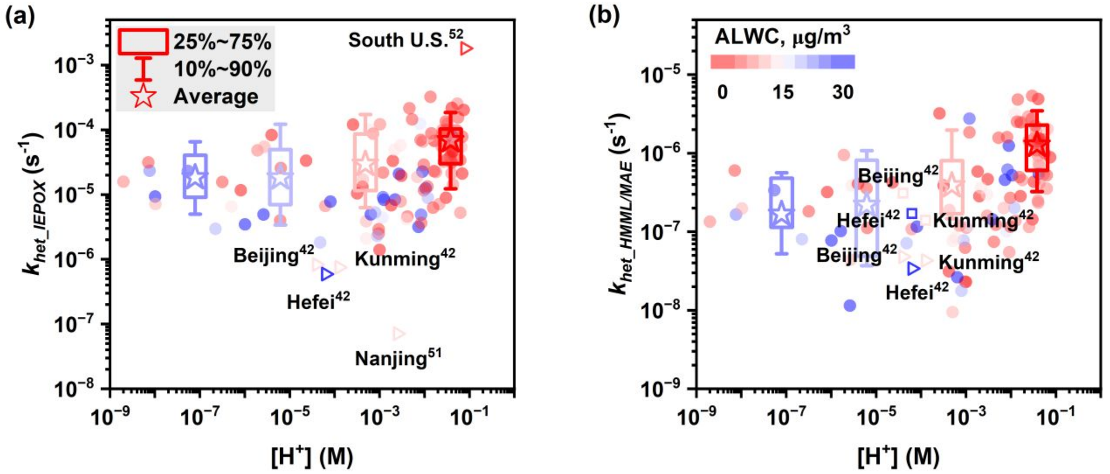

Figure S24. Heterogeneous reaction rate coefficients (k_{het}) of (a) IEPOX and (b) HMML/MAE as a function of aerosol liquid water content (ALWC). The data points and boxes are colored by aerosol acidity ($[H^+]$). The triangle points were values estimated directly with parameters derived from bulk solution experiments. The square points were values of those triangle points constrained by observation data.

Table S6. Uncertainties in multivariate linear regression (MLR) analysis[a] based on 1000 bootstrap samples.

Factors		Coefficients	Bias	Standard error	Significance (2-tailed)	95% confidence interval	
						Lower	Upper
IEPOX	$k_{H_2O,H^+}H_{aq}$	58446.2	-11719.5	13634.5	0.025	20001.1	73448.5
	$k_{H_2O,HSO_4^-}H_{aq}$	211182.2	5243.7	24370.2	0.009	168633.5	264194.5
	$k_{SO_4^{2-},H^+}H_{aq}$	66671.9	-11148.2	14562.3	0.014	26780.1	83866.2
HMML/MAE	$k_{H_2O,H^+}H_{aq}$	1544.6	745.2	775.3	0.022	765.7	3811.3
	$k_{H_2O,HSO_4^-}H_{aq}$	3397.3	294.2	756.8	0.013	2209.4	5170.3
	$k_{SO_4^{2-},H^+}H_{aq}$	731.5	-77.7	271.4	0.020	121.3	1187.1

[a] MLR analysis was performed with IBM SPSS Statistics 20.0 software.

Table S7. Comparisons of acid-catalyzed aqueous-phase reaction constants for IEPOX and HMML/MAE estimated with original output of ISORROPIA-II model (Base case) and with [H^+] or ALWC increased or decreased by 20%.

Reference	IEPOX (M^{-1} atm^{-1} s^{-1})			HMML/MAE (M^{-1} atm^{-1} s^{-1})		
	$k_{H_2O,H^+}H_{aq}$	$k_{H_2O,HSO_4^-}H_{aq}$	$k_{SO_4^{2-},H^+}H_{aq}$	$k_{H_2O,H^+}H_{aq}$	$k_{H_2O,HSO_4^-}H_{aq}$	$k_{SO_4^{2-},H^+}H_{aq}$
Base case	$(5.9\pm1.3) \times 10^4$	$(2.1\pm0.3) \times 10^5$	$(6.7\pm1.3) \times 10^4$	$(1.5\pm0.8) \times 10^3$	$(3.4\pm0.7) \times 10^3$	$(7.3\pm2.7) \times 10^2$
[H^+] increased by 20%	$(4.9\pm1.0) \times 10^4$	$(2.1\pm0.2) \times 10^5$	$(6.7\pm1.3) \times 10^4$	$(1.2\pm0.7) \times 10^3$	$(3.4\pm0.6) \times 10^3$	$(7.3\pm2.7) \times 10^2$
[H^+] decreased by 20%	$(7.3\pm1.6) \times 10^4$	$(2.1\pm0.3) \times 10^5$	$(6.7\pm1.4) \times 10^4$	$(1.9\pm1.0) \times 10^3$	$(3.4\pm0.7) \times 10^3$	$(7.3\pm2.7) \times 10^2$
ALWC increased by 20%	$(7.1\pm1.5) \times 10^4$	$(2.5\pm0.6) \times 10^5$	$(8.0\pm1.6) \times 10^4$	$(1.8\pm1.0) \times 10^3$	$(4.1\pm0.8) \times 10^3$	$(8.8\pm3.2) \times 10^2$
ALWC decreased by 20%	$(4.7\pm0.9) \times 10^4$	$(1.7\pm0.2) \times 10^5$	$(5.4\pm1.0) \times 10^4$	$(1.2\pm0.6) \times 10^3$	$(2.7\pm0.6) \times 10^3$	$(5.8\pm2.1) \times 10^2$

References

(1) He, X.; Wang, Q.; Huang, X.H.H.; Huang, D.D.; Zhou, M.; Qiao, L.; Zhu, S.; Ma, Y.; Wang, H.; Li, L.; Huang, C.; Xu, W.; Worsnop, D.R.; Goldstein, A.H.; Yu, J.Z. Hourly measurements of organic molecular markers in urban Shanghai, China: Observation of enhanced formation of secondary organic aerosol during particulate matter episodic periods. *Atmospheric environment (1994)* **2020,** *240*, 117807; 10.1016/j.atmosenv.2020.117807.

(2) Wang, Q.; He, X.; Zhou, M.; Huang, D.D.; Qiao, L.; Zhu, S.; Ma, Y.; Wang, H.; Li, L.; Huang, C.; Huang, X.H.H.; Xu, W.; Worsnop, D.; Goldstein, A.H.; Guo, H.; Yu, J.Z. Hourly Measurements of Organic Molecular Markers in Urban Shanghai, China: Primary Organic Aerosol Source Identification and Observation of Cooking Aerosol Aging. *ACS Earth Space Chem* **2020,** *4* (9), 1670-1685; 10.1021/acsearthspacechem.0c00205.

(3) Zhu, S.; Wang, Q.; Qiao, L.; Zhou, M.; Wang, S.; Lou, S.; Huang, D.; Wang, Q.; Jing, S.; Wang, H.; Chen, C.; Huang, C.; Yu, J.Z. Tracer-based characterization of source variations of PM 2.5 and organic carbon in Shanghai influenced by the COVID-19 lockdown. *Faraday Discuss.* **2021,** *226*, 112-137; 10.1039/D0FD00091D.

(4) Li, L.; Chao, B.; Wang, W.; Luo, Y.; Wang, L.; Lin, L.; Yang, G.; Wu, J. Identification and quantification of IEPOX in ambient aerosols, using electron and chemical ionization sources GC/MS as their trimethylsilyl ethers, and using H-NMR. *Sci. Total Environ.* **2023,** *872*, 162186; 10.1016/j.scitotenv.2023.162186.

(5) Stone, E. A.; Nguyen, T. T.; Pradhan, B. B.; Man Dangol, P. Assessment of biogenic secondary organic aerosol in the Himalayas. Environ. Chem. **2012**, *9*, (3), 263-272.

(6) Shen RQ, Ding X, He QF, et al. Seasonal variation of secondary organic aerosol tracers in Central Tibetan Plateau[J]. Atmospheric Chemistry and Physics: 2015, 15: 8781-8793.

(7) Lin, Y.; Zhang, Z.; Docherty, K.S.; Zhang, H.; Budisulistiorini, S.H.; Rubitschun, C.L.; Shaw, S.L.; Knipping, E.M.; Edgerton, E.S.; Kleindienst, T.E.; Gold, A.; Surratt, J.D. Isoprene Epoxydiols as Precursors to Secondary Organic Aerosol Formation: Acid-Catalyzed Reactive Uptake Studies with Authentic Compounds. *Environ. Sci. Technol.* **2012,** *46* (1), 250-258; 10.1021/es202554c.

(8) Worton, D.R.; Surratt, J.D.; LaFranchi, B.W.; Chan, A.W.H.; Zhao, Y.; Weber, R.J.; Park, J.; Gilman, J.B.; de Gouw, J.; Park, C.; Schade, G.; Beaver, M.; Clair, J.M.S.; Crounse, J.; Wennberg, P.; Wolfe, G.M.; Harrold, S.; Thornton, J.A.; Farmer, D.K.; Docherty, K.S.; Cubison, M.J.; Jimenez, J.; Frossard, A.A.; Russell, L.M.; Kristensen, K.; Glasius, M.; Mao, J.; Ren, X.; Brune, W.; Browne, E.C.; Pusede, S.E.; Cohen, R.C.; Seinfeld, J.H.; Goldstein, A.H. Observational Insights into Aerosol Formation from Isoprene. *Environ. Sci. Technol.* **2013,** *47* (20), 11403-11413; 10.1021/es4011064.

(9) Li, L.; Chao, B.; Wang, W.; Luo, Y.; Wang, L.; Lin, L.; Yang, G.; Wu, J. Identification and quantification of IEPOX in ambient aerosols, using electron and chemical ionization sources GC/MS as their trimethylsilyl ethers, and using H-NMR. *Sci. Total Environ.* **2023,** *872*, 162186; 10.1016/j.scitotenv.2023.162186.

(10) Beaver, M.R.; Clair, J.M.S.; Paulot, F.; Spencer, K.M.; Crounse, J.D.; LaFranchi, B.W.; Min, K.E.; Pusede, S.E.; Wooldridge, P.J.; Schade, G.W.; Park, C.; Cohen, R.C.; Wennberg, P.O. Importance of biogenic precursors to the budget of organic nitrates: observations of multifunctional organic nitrates by CIMS and TD-LIF during BEARPEX 2009. *Atmospheric chemistry and physics* **2012,** *12* (13), 5773-5785; 10.5194/acp-12-5773-2012.

(11) Brown, S.S.; deGouw, J.A.; Warneke, C.; Ryerson, T.B.; Dubé, W.P.; Atlas, E.; Weber, R.J.; Peltier, R.E.; Neuman, J.A.; Roberts, J.M.; Swanson, A.; Flocke, F.; McKeen, S.A.; Brioude, J.; Sommariva, R.; Trainer, M.; Fehsenfeld, F.C.; Ravishankara, A.R. Nocturnal isoprene oxidation over the Northeast United States in summer and its impact on reactive nitrogen partitioning and secondary organic aerosol. *Atmospheric chemistry and physics* **2009,** *9* (9), 3027-3042; 10.5194/acp-9-3027-2009.

(12) Fiore, A.M.; Horowitz, L.W.; Purves, D.W.; Levy II, H.; Evans, M.J.; Wang, Y.; Li, Q.; Yantosca, R.M. Evaluating the contribution of changes in isoprene emissions to surface ozone trends over the eastern United States. *Journal of Geophysical Research: Atmospheres* **2005,** *110*, D12303; 10.1029/2004JD005485.

(13) Starn, T.K.; Shepson, P.B.; Bertman, S.B.; Riemer, D.D.; Zika, R.G.; Olszyna, K. Nighttime isoprene chemistry at an urban-impacted forest site. *Journal of Geophysical Research: Atmospheres* **1998,** *103*, 22437-22447; 10.1029/98JD01201.

(14) Zhu, W.; Zhou, M.; Cheng, Z.; Yan, N.; Huang, C.; Qiao, L.; Wang, H.; Liu, Y.; Lou, S.; Guo, S. Seasonal variation of aerosol compositions in Shanghai, China: Insights from particle aerosol mass spectrometer observations. *Sci. Total Environ.* **2021,** *771*, 144948; 10.1016/j.scitotenv.2021.144948.

(15) Williams, B.J.; Goldstein, A.H.; Kreisberg, N.M.; Hering, S.V. In situ measurements of gas/particle-phase transitions for atmospheric semivolatile organic compounds. *Proc. Natl. Acad. Sci. U. S. A.* **2010,** *107* (15), 6676-6681; 10.1073/pnas.0911858107.
(16) Zhang, J.; Liu, J.; Ding, X.; He, X.; Zhang, T.; Zheng, M.; Choi, M.; Isaacman-VanWertz, G.; Yee, L.; Zhang, H.; Misztal, P.; Goldstein, A.H.; Guenther, A.B.; Budisulistiorini, S.H.; Surratt, J.D.; Stone, E.A.; Shrivastava, M.; Wu, D.; Yu, J.Z.; Ying, Q. New formation and fate of Isoprene SOA markers revealed by field data-constrained modeling. *NPJ climate and atmospheric science* **2023,** *6* (1), 69-8; 10.1038/s41612-023-00394-3.
(17) Zhou, M.; Zheng, G.; Wang, H.; Qiao, L.; Zhu, S.; Huang, D.; An, J.; Lou, S.; Tao, S.; Wang, Q.; Yan, R.; Ma, Y.; Chen, C.; Cheng, Y.; Su, H.; Huang, C. Long-term trends and drivers of aerosol pH in eastern China. *Atmos. Chem. Phys.* **2022,** *22* (20), 13833-13844; 10.5194/acp-22-13833-2022.
(18) Guo, H.; Xu, L.; Bougiatioti, A.; Cerully, K.M.; Capps, S.L.; Hite Jr., J.R.; Carlton, A.G.; Lee, S.-.; Bergin, M.H.; Ng, N.L.; Nenes, A.; Weber, R.J. Fine-particle water and pH in the southeastern United States. *Atmos. Chem. Phys.* **2015,** *15* (9), 5211-5228; 10.5194/acp-15-5211-2015.
(19) Zhang, Y.; Ding, X.; He, Q.; Wen, T.; Wang, J.; Yang, K.; Jiang, H.; Cheng, Q.; Liu, P.; Wang, Z.; He, Y.; Hu, W.; Wang, Q.; Xin, J.; Wang, Y.; Wang, X. Observational Insights into Isoprene Secondary Organic Aerosol Formation through the Epoxide Pathway at Three Urban Sites from Northern to Southern China. *Environ. Sci. Technol.* **2022,** *56* (8), 4795-4805; 10.1021/acs.est.1c06974.
(20) He, Q.; Ding, X.; Fu, X.; Zhang, Y.; Wang, J.; Liu, Y.; Tang, M.; Wang, X.; Rudich, Y. Secondary Organic Aerosol Formation From Isoprene Epoxides in the Pearl River Delta, South China: IEPOX- and HMML-Derived Tracers. *Journal of geophysical research: Atmospheres* **2018,** *123* (13), 6999-7012; 10.1029/2017JD028242.
(21) Budisulistiorini, S.H.; Li, X.; Bairai, S.T.; Renfro, J.; Liu, Y.; Liu, Y.J.; McKinney, K.A.; Martin, S.T.; McNeill, V.F.; Pye, H.O.T.; Nenes, A.; Neff, M.E.; Stone, E.A.; Mueller, S.; Knote, C.; Shaw, S.L.; Zhang, Z.; Gold, A.; Surratt, J.D. Examining the effects of anthropogenic emissions on isoprene-derived secondary organic aerosol formation during the 2013 Southern Oxidant and Aerosol Study (SOAS) at the Look Rock, Tennessee ground site. *Atmospheric chemistry and physics* **2015,** *15* (15), 8871-8888; 10.5194/acp-15-8871-2015.
(22) Rattanavaraha, W.; Chu, K.; Budisulistiorini, S.H.; Riva, M.; Ying-Hsuan Lin; Edgerton, E.S.; Baumann, K.; Shaw, S.L.; Guo, H.; King, L.; Weber, R.J.; Neff, M.E.; Stone, E.A.; Offenberg, J.H.; Zhang, Z.; Gold, A.; Surratt, J.D. Assessing the impact of anthropogenic pollution on isoprene-derived secondary organic aerosol formation in $PM_{2.5}$ collected from the Birmingham, Alabama, ground site during the 2013 Southern Oxidant and Aerosol Study. *Atmospheric chemistry and physics* **2016,** *16* (8), 4897-4914; 10.5194/acp-16-4897-2016.
(23) Lin, Y.; Zhang, Z.; Docherty, K.S.; Zhang, H.; Budisulistiorini, S.H.; Rubitschun, C.L.; Shaw, S.L.; Knipping, E.M.; Edgerton, E.S.; Kleindienst, T.E.; Gold, A.; Surratt, J.D. Isoprene Epoxydiols as Precursors to Secondary Organic Aerosol Formation: Acid-Catalyzed Reactive Uptake Studies with Authentic Compounds. *Environ. Sci. Technol.* **2012,** *46* (1), 250-258; 10.1021/es202554c.
(24) Cui, T.; Zeng, Z.; Dos Santos, E.,O.; Zhang, Z.; Chen, Y.; Zhang, Y.; Rose, C.A.; Budisulistiorini, S.H.; Collins, L.B.; Bodnar, W.M.; de Souza, R.,A.F.; Martin, S.T.; Machado, C.M.D.; Turpin, B.J.; Gold, A.; Ault, A.P.; Surratt, J.D. Development of a hydrophilic interaction liquid chromatography (HILIC) method for the chemical characterization of water-soluble isoprene epoxydiol (IEPOX)-derived secondary organic aerosol. *Environ Sci Process Impacts* **2018,** *20* (11), 1524-1536; 10.1039/c8em00308d.
(25) Zhang, L.; Fang, G.C.; Liu, C.K.; Huang, Y.L.; Huang, J.H.; Huang, C.S. Dry deposition fluxes and deposition velocities of seven trace metal species at five sites in central Taiwan – a summary of surrogate surface measurements and a comparison with model estimations. *Atmospheric chemistry and physics* **2012,** *12* (7), 3405–3417; 10.5194/acp-12-3405-2012.
(26) Poor, N.; Tremblay, R.; Kay, H.; Bhethanabotla, V.; Swartz, E.; Luther, M.; Campbell, S. Atmospheric concentrations and dry deposition rates of polycyclic aromatic hydrocarbons (PAHs) for Tampa Bay, Florida, USA. *Atmospheric environment (1994)* **2004,** *38* (35), 6005–6015; 10.1016/j.atmosenv.2004.06.037.
(27) Zufall, M.J.; Davidson, C.I.; Caffrey, P.F.; Ondov, J.M. Airborne Concentrations and Dry Deposition Fluxes of Particulate Species to Surrogate Surfaces Deployed in Southern Lake Michigan. *Environ. Sci. Technol.* **1998,** *32* (11), 1623–1628; 10.1021/es9706458.
(28) Eddingsaas, N.C.; VanderVelde, D.G.; Wennberg, P.O. Kinetics and Products of the Acid-Catalyzed Ring-Opening of Atmospherically Relevant Butyl Epoxy Alcohols. *J Phys Chem A* **2010,** 114 (31), 8106-8113; 10.1021/jp103907c.

(29) Zhang, Y.; Tang, L.; Sun, Y.; Favez, O.; Canonaco, F.; Albinet, A.; Couvidat, F.; Liu, D.; Jayne, J.T.; Wang, Z.; Croteau, P.L.; Canagaratna, M.R.; Zhou, H.; Prévôt, A.S.H.; Worsnop, D.R. Limited formation of isoprene epoxydiols-derived secondary organic aerosol under NO_x-rich environments in Eastern China. *Geophys. Res. Lett.* **2017,** *44* (4), 2035-2043; 10.1002/2016GL072368.
(30) Borbon, A.; Gilman, J.B.; Kuster, W.C.; Grand, N.; Chevaillier, S.; Colomb, A.; Dolgorouky, C.; Gros, V.; Lopez, M.; Sarda-Esteve, R.; Holloway, J.; Stutz, J.; Petetin, H.; McKeen, S.; Beekmann, M.; Warneke, C.; Parrish, D.D.; de Gouw, J.A. Emission ratios of anthropogenic volatile organic compounds in northern mid-latitude megacities: Observations versus emission inventories in Los Angeles and Paris. *Journal of geophysical research.Atmospheres* **2013,** *118* (4), 2041–2057; 10.1002/jgrd.50059.
(31) Gouw, J.A.; Gilman, J.B.; Kim, S.-; Alvarez, S.L.; Dusanter, S.; Graus, M.; Griffith, S.M.; Isaacman-VanWertz, G.; Kuster, W.C.; Lefer, B.L.; Lerner, B.M.; McDonald, B.C.; Rappenglück, B.; Roberts, J.M.; Stevens, P.S.; Stutz, J.; Thalman, R.; Veres, P.R.; Volkamer, R.; Warneke, C.; Washenfelder, R.A.; Young, C.J. Chemistry of Volatile Organic Compounds in the Los Angeles Basin: Formation of Oxygenated Compounds and Determination of Emission Ratios. *Journal of geophysical research.Atmospheres* **2018,** *123* (4), 2298–2319; 10.1002/2017JD027976.
